# The zebrafish cationic amino acid transporter/glycoprotein-associated family: sequence and spatiotemporal distribution during development of the transport system b^0,+^ (*slc3a1*/*slc7a9*)

**DOI:** 10.1007/s10695-021-00984-z

**Published:** 2021-08-02

**Authors:** Ståle Ellingsen, Shailesh Narawane, Anders Fjose, Tiziano Verri, Ivar Rønnestad

**Affiliations:** 1 Department of Molecular Biology, University of Bergen, Postbox 7803, N-5020 Bergen, Norway; 2 Present Address: Department of Biological Sciences, University of Bergen, Postbox 7803, N-5020 Bergen, Norway; 3 Department of Biological and Environmental Sciences and Technologies, University of Salento, via Prov.le Lecce-Monteroni, 73100 Lecce, Italy

**Keywords:** Gut, Heteromeric amino acid transporters, Kidney, Proximal convoluted tubule, Proximal straight tubule

## Abstract

System b^0,+^ absorbs lysine, arginine, ornithine, and cystine, as well as some (large) neutral amino acids in the mammalian kidney and intestine. It is a heteromeric amino acid transporter made of the heavy subunit SLC3A1/rBAT and the light subunit SLC7A9/b^0,+^AT. Mutations in these two genes can cause cystinuria in mammals. To extend information on this transport system to teleost fish, we focused on the *slc3a1* and *slc7a9* genes by performing comparative and phylogenetic sequence analysis, investigating gene conservation during evolution (synteny), and defining early expression patterns during zebrafish (*Danio rerio*) development. Notably, we found that *slc3a1* and *slc7a9* are non-duplicated in the zebrafish genome. Whole-mount in situ hybridization detected co-localized expression of *slc3a1* and *slc7a9* in pronephric ducts at 24 h post-fertilization and in the proximal convoluted tubule at 3 days post-fertilization (dpf). Notably, both the genes showed co-localized expression in epithelial cells in the gut primordium at 3 dpf and in the intestine at 5 dpf (onset of exogenous feeding). Taken together, these results highlight the value of *slc3a1* and *slc7a9* as markers of zebrafish kidney and intestine development and show promise for establishing new zebrafish tools that can aid in the rapid screening(s) of substrates. Importantly, such studies will help clarify the complex interplay between the absorption of dibasic amino acids, cystine, and (large) neutral amino acids and the effect(s) of such nutrients on organismal growth.

**Supplementary Information:**

The online version contains supplementary material available at 10.1007/s10695-021-00984-z.

## Introduction

In humans, the solute carrier 7 (SLC7) family consists of 15 members (Fotiadis et al. 2013). The encoded proteins are divided into two subgroups: cationic amino acid transporters and light subunits of heterodimeric amino acid transporters (HATs). HATs are characterized by broad substrate specificity toward several amino acid classes: neutral (SLC7A5, SLC7A8, SLC7A10, and SLC7A12), aromatic (SLC7A15), negatively charged (SLC7A11 and SLC7A13), and cationic plus neutral (SLC7A6, SLC7A7, and SLC7A9) amino acids (Chillaron et al. 2001; Bröer 2008; Fotiadis et al. 2013; Kandasamy et al. 2018). Each HAT consists of two subunits: light subunit and heavy subunit. Belonging to the solute carrier 3 (SLC3) family, the heavy subunit is a membrane glycoprotein that consists of a single transmembrane domain and a large extracellular domain. The subunits interact via a disulfide bridge between the two cysteine residues of the proteins forming HATs. The SLC3 family consists of two heavy chain members (SLC3A1 and SLC3A2) (Fotiadis et al. 2013), which are involved in the trafficking of the heteromeric complex to the plasma membrane, while light chains specifically catalyze the transport process (Bröer et al. 2001; Wagner et al. 2001; Chillaron et al. 2001; Bröer 2008; Fotiadis et al. 2013; Kandasamy et al. 2018).

HATs play important roles in membrane and cell transport. They play a key role in absorbing dietary proteins, with various transporters present on the apical and basolateral membranes of intestinal epithelial cells, to absorb amino acids from the intestinal lumen and release them into blood (Chillaron et al. 2001; Bröer 2008; Fotiadis et al. 2013; Kandasamy et al. 2018). HATs also have important functions in the kidney, where they are involved in the adjustment of amino acid levels in the ultrafiltrate and final urine (Chillaron et al. 2001; Bröer 2008; Fotiadis et al. 2013; Kandasamy et al. 2018). One such amino acid transport system is system b^0,+^, which consists of the subunits SLC3A1 and SLC7A9, otherwise known as rBAT and b^0,+^AT, respectively (Bröer 2008). System b^0,+^ (SLC3A1/SLC7A9) induces high-affinity, Na^+^-independent transport of lysine, arginine, ornithine (dibasic), and cysteine (neutral) amino acids in the kidney and intestine (Bertran et al. 1992, 1993; Magagnin et al. 1992; Lee et al. 1993; Palacin 1994; Chairoungdua et al. 1999; Mizoguchi et al. 2001; Pfeiffer et al. 1999). *SLC3A1* mRNA is strongly expressed in the rabbit kidney and intestinal mucosa (Bertran et al. 1992; Magagnin et al. 1992). The rabbit *SLC3A1* encodes a 77.8-KDa protein of 677 amino acids, with a single predicted transmembrane region (Bertran et al. 1992). *SLC7A9*, which was first isolated from humans and rats, encodes a 40 kDa protein of 487 amino acids with 12 predicted transmembrane regions, and forms a heterodimer with SLC3A1 via a disulfide bridge (Chairoungdua et al. 1999; Pfeiffer et al. 1999). Initial studies showed that SLC7A9 and SLC3A1 co-localize to the apical membrane of the renal proximal tubule (Chairoungdua et al. 1999; Mizoguchi et al. 2001; Mora et al. 1996; Pfeiffer et al. 1999) and small intestinal epithelium (Dave et al. 2004).

SLC7A9/SLC3A1 collaborates with other systems (SLC7A7/SLC3A2 or SLC7A6/SLC3A2) for cystine and cationic amino acid transepithelial reabsorption; parallel and unidirectional neutral amino acid transport is required for this reabsorption (Bauch et al. 2003).

Initially, SLC3A1 was found to induce the exchange of dibasic (inward) with neutral (outward) amino acids through the membrane (Busch et al. 1994). Later, it was shown that the cytoplasmic tail and transmembrane region of SLC3A1 collectively play an important role in its functional interaction with SLC7A9 (Franca et al. 2005). SLC3A1 is quickly degraded in the absence of SLC7A9, while SLC7A9 is stable in the absence of SLC3A1 (Bauch and Verrey 2002). Both subunits depend on each other for apical surface expression, and when co-expressed, they link covalently and yield a fully glycosylated and more stable SLC3A1 (Bauch and Verrey 2002). SLC7A9 alone is adequate to catalyze transport (Reig et al. 2002), but there is also evidence that mutations in *SLC3A1* may affect the transport properties of system b^0,+^ (Pineda et al. 2004).

Mutations in *SLC3A1* and *SLC7A9* cause cystinuria (Calonge et al. 1995; Palacin 1994; Miyamoto et al. 1995; Bisceglia et al. 2001; Egoshi et al. 2000; Font et al. 2001; Dello Strologo et al. 2002). The clinical symptoms of cystinuria are related to nephrolithiasis, due to the precipitation of cystine in urine. Mutations in *SLC3A1* cause cystinuria type A, whereas mutations in *SLC7A9* cause cystinuria type B (Dello Strologo et al. 2002). When the orthologous genes were knocked out in mice, the mutant displayed phenotypes similar to those in humans (Feliubadalo et al. 2003; Peters et al. 2003). Moreover, when a type AB cystinuria mouse model was generated upon crossing *Slc3a1*^*−/−*^ mice with *Slc7a9*^*−/−*^ mice, the double heterozygous mice *(Slc7a9*^*+/-*^*/Slc3a1*^*+/-*^) exhibited lower expression of system b^0,+^ and higher hyperexcretion of cystine than the single heterozygotes (*Slc7a9*^*+/-*^*/Slc3a1*^*+/+*^ and *Slc7a9*^*+/+*^/Slc3a1^*+/-*^), giving rise to lithiasis, thus showing that cystinuria has a digenic inheritance in this mouse model (Espino et al. 2015).

Information on Slc3a1 and Slc7a9 proteins in teleost fish is limited to a few species and organ systems. For instance, in adult Mozambique tilapia (*Oreochromis mossambicus*) intestine, differential mRNA expression of *slc3a1* and *slc7a9* was detected along the gastrointestinal tract. This expression is variably affected by water salinity (Nitzan et al. 2017), degree of completion of the food digestion process (Nitzan et al. 2017), and growth hormones (Petro-Sakuma et al. 2020). In addition, in turbot (*Scophthalmus maximus*) primary muscle cells, *slc7a9* mRNA expression has been shown to decrease post soy phosphatidic acid administration (Wang et al. 2018). To date, very little is known about amino acid transporters in zebrafish (*Danio rerio*), despite the increasing relevance of this teleost fish model. In an attempt to fill these gaps, we hereby report a study on the system b^0,+^ in zebrafish, with emphasis on sequence analysis and spatiotemporal distribution in the developing kidney and gut. The gene identity of zebrafish vs. other teleost and higher vertebrate (human included) counterparts was assessed by means of synteny. The amino acid sequences of the proteins composing the zebrafish system b^0,+^ were validated by comparison with their orthologs in vertebrates and construction of a phylogenetic tree. In situ hybridization at embryonic and early larval stages revealed segment-specific expression of *slc3a1* and *slc7a9* in the nephron. In addition, expression was observed in the gut, where the transcripts were localized to the intestinal epithelial cells. Taken together, these findings extend our knowledge of system b^0,+^ in teleost fish by identifying the expression patterns of its components *slc3a1* and *slc7a9* in larval zebrafish. Thus, they serve as a starting point to fully clarify the role(s) of *slc3a1* and *slc7a9* in the development and function of the teleost fish kidney and intestine.

## Material and methods

### Zebrafish

Zebrafish were maintained and bred at the University of Bergen, as described elsewhere (Stuart et al. 1988). Zebrafish embryos were obtained via natural mating, and pigmentation was prevented by addition of 0.003% phenylthiourea to the E3 medium.

### Sequence analysis

The nucleotide and protein sequences used in this study were obtained from Ensembl (http://www.ensembl.org/index.html). The predicted transcripts used for generating the in situ probes were Ensembl Transcript ID: ENSDART00000132393 (Ensembl gene ID: ENSDARG00000017165) for *slc3a1* and Ensembl Transcript ID: ENSDART00000100479 (Ensembl gene ID: ENSDARG00000005894) for *slc7a9*.

The SLC3A1/Slc3a1- and SLC7A9/Slc7a9-type amino acid sequences used for sequence comparison are shown in Table S1 (both Ensembl and UniProtKB peptide IDs reported; see also Appendix I) for each selected vertebrate species. Multiple sequence alignments were performed on orthologous proteins using ClustalO (https://www.ebi.ac.uk/Tools/msa/clustalo/) (Sievers et al. 2011), and neighbor-joining method-based phylogenetic trees were constructed using MEGA7 (http://www.megasoftware.net).

Putative transmembrane domains were predicted using TMHMM 2.0 (http://www.cbs.dtu.dk/services/TMHMM/), which is part of the Simple Modular Architecture Research Tool (SMART; http://smart.embl-heidelberg.de/). Other domains were predicted using PFAM 32.0 (http://pfam.xfam.org/), which was also implemented in SMART. Potential N-glycosylation, cyclic adenosine monophosphate/cyclic guanosine monophosphate dependent protein kinase, and protein kinase C recognition sequences were identified using PROSITE 19.7 computational tools (http://www.expasy.org/prosite/).

Syntenic conservation of the zebrafish *slc3a1* and *slc7a9* genes with respect to other vertebrate orthologous genes was assessed using Genomicus (database version: 93.01; Nguyen et al. 2018) available online at http://genomicus.biologie.ens.fr/genomicus-93.01/cgi-bin/search.pl using the search terms ‘slc3a1’ and ‘slc7a9’, respectively.

### Protein modeling

**Fig. 1:**
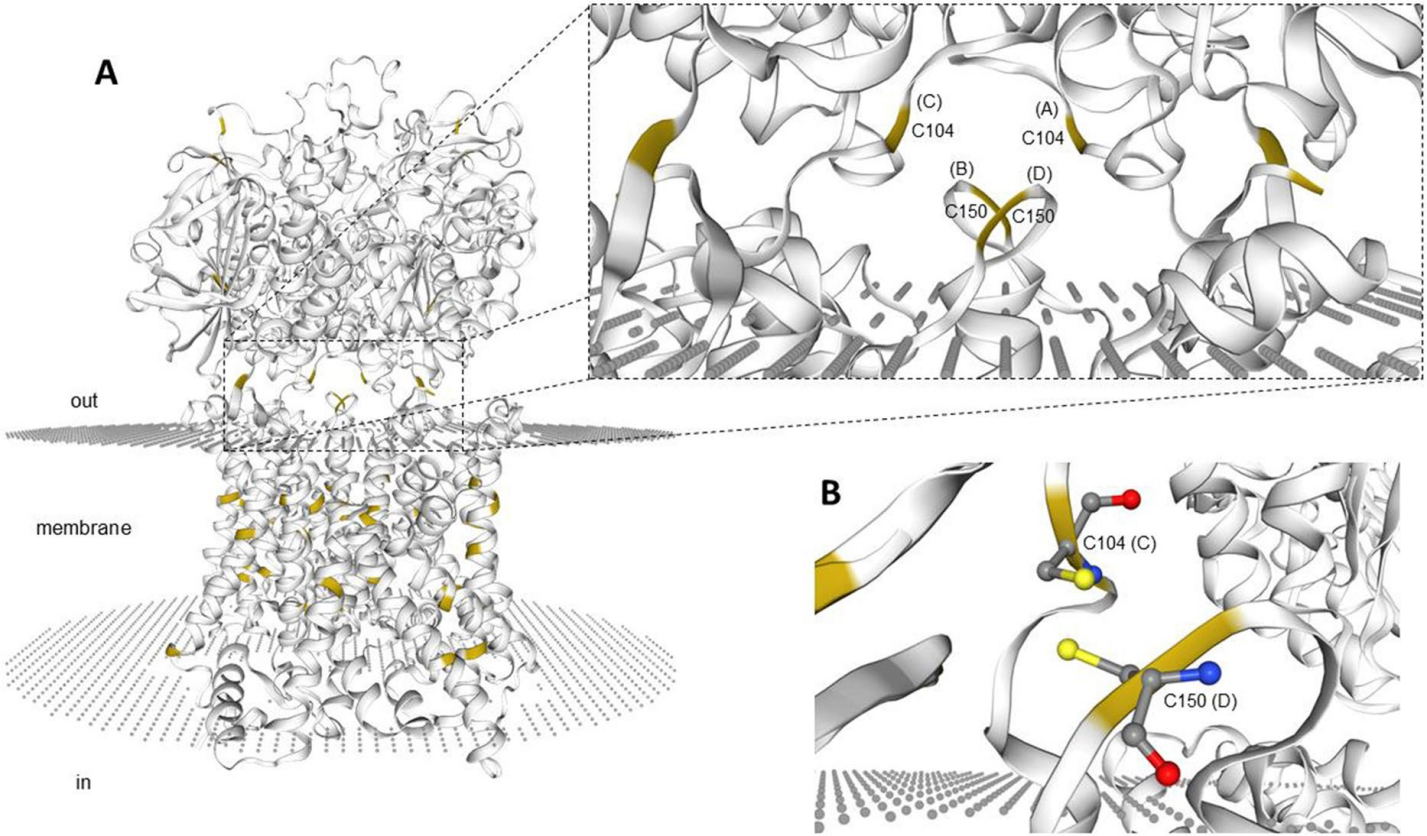
(**A**) Three-dimensional appearance (lateral view) of the zebrafish Slc7a9(b^0,+^AT)-Slc3a1(rBAT) complex bound to arginine in the hetero-2–2-mer (i.e., heterotetramer made of 2 heterodimers) form and (**B**) snapshot of the putative disulfide bridge linking covalently one of the two heterodimers (i.e., the heterodimer made of chain C, Slc3a1 and chain D, Slc7a9). The model was built using Protein Data Bank Acc. No. 6li9.1 as a template. Cysteine residues only are highlighted (yellow). A, chain A; B, chain B; C, chain C; D, chain D. Chains A and C refer to zebrafish Slc3a1; chains B and D refer to zebrafish Slc7a9. C104, cysteine residue C^104^ on chain C, Slc3a1; C150, cysteine residue C^150^ on chain D, Slc7a9

SWISS-MODEL (Waterhouse et al. 2018) in the “Add Hetero Target” mode (https://swissmodel.expasy.org/interactive) was used to predict the structure of the zebrafish Slc3a1 and Slc7a9 proteins using their primary sequences (UniProtKB peptide IDs: F1QEA9 and F1QGJ2, respectively; see Table S1). The search for templates directly targeted the human heteromeric amino acid transporter b^0,+^AT-rBAT complex bound to the arginine structure (Protein Data Bank Acc. No.: 6li9.1) (Yan et al. 2020) in the hetero-2–2-mer (i.e*.*, heterotetramer made of two heterodimers) form (for details, see Fig. 1A; see also Fig. S1, Fig. S2 and Appendix I in the Supplementary Materials I), which was used as a template to build the model. SWISS-MODEL view tools were also used to visualize the final three-dimensional structure (Fig. 1).

### RNA probe preparation, DIG-labeling, and in situ hybridization

The in situ probes for *slc3a1* and *slc7a9* genes were isolated using PCR amplification with the primers listed in Table S2. The PCR amplicons were cloned into the pCRII-TOPO vector (Invitrogen, Carlsbad, USA). The obtained sequences were verified using sequencing.

Plasmid DNA was linearized with appropriate restriction endonucleases for 5 h at 37 °C, purified using the QIAquick® Nucleotide Removal Kit (Qiagen, Hilden, Germany), following which the degree of linearization was examined on a 1% agarose gel. In vitro transcription to produce digoxygenin (DIG)-labeled RNA probe was carried out as follows: linearized plasmids, 1 µg; DIG labeling mix (Roche, Mannheim, Germany) 2 µl; transcription buffer, 2 µl; RNase inhibitor (Roche), 1 µl; T7/Sp6 RNA polymerase (Roche), 2 µl; RNase-free double distilled H_2_O were combined to a final volume of 20 µl. The mixture was incubated at 37 °C for 2 h. This was followed by DNase I treatment for 15 min at 37 °C. Labeled RNA was purified using the RNeasy® Mini Kit (Qiagen), following which its probe length was verified using agarose gel electrophoresis. It was then dissolved in 150 µl hybridization buffer and stored at -20 °C until use.

Whole-mount in situ hybridization was performed as previously described (Seo et al. 1998). Briefly, DIG-labeled antisense and sense (negative control) zebrafish *slc7a9* and *slc3a1* RNA probes were used. Embryos were fixed in 4% glutaraldehyde in 1 × phosphate-buffered saline (PBS) and stained using nitro-blue tetrazolium chloride and 5-bromo-4-chloro-3'-indolyphosphate. For cryo cross-sections, the embryos stained using the in situ hybridization protocol were embedded in Tissue-Tek® (Sakura Finetek, Zoeterwoude, The Netherlands) after overnight incubation in 25% sucrose in 1 × PBS. Sections (20 µm thick) were cut using a CM1800 Cryostat (Leica, Wetzlar, Germany) at -20 °C.

### Imaging

In situ hybridization images were captured with a Leica M420™ and Nikon EPI-FL3™ microscope equipped with a micropublisher 5.0 RTV camera (QImaging, Tucson, AZ, USA). Figures were generated using CS2 Photoshop™ and Illustrator™ (Adobe San Jose, CA, USA).

### Ethical treatment of animals

Fish were maintained and the experiments were conducted in compliance with the Norwegian Animal Welfare Act guidelines. No ethical permission was needed to be obtained for this study. According to the EU Directive 2010/63/EU on the protection of animals used for scientific purposes, implemented in Norwegian legislation as of 12.12.2014, early life stages of zebrafish are not protected as animals until they are capable of independent feeding, which is, 5 days post fertilization (dpf).

## Results

### Zebrafish system b^0+^ is conserved among vertebrates

Detailed sequence analysis and interspecies comparison among vertebrates were performed to ascertain the identity of the predicted zebrafish system b^0+^ components.

**Fig. 2:**
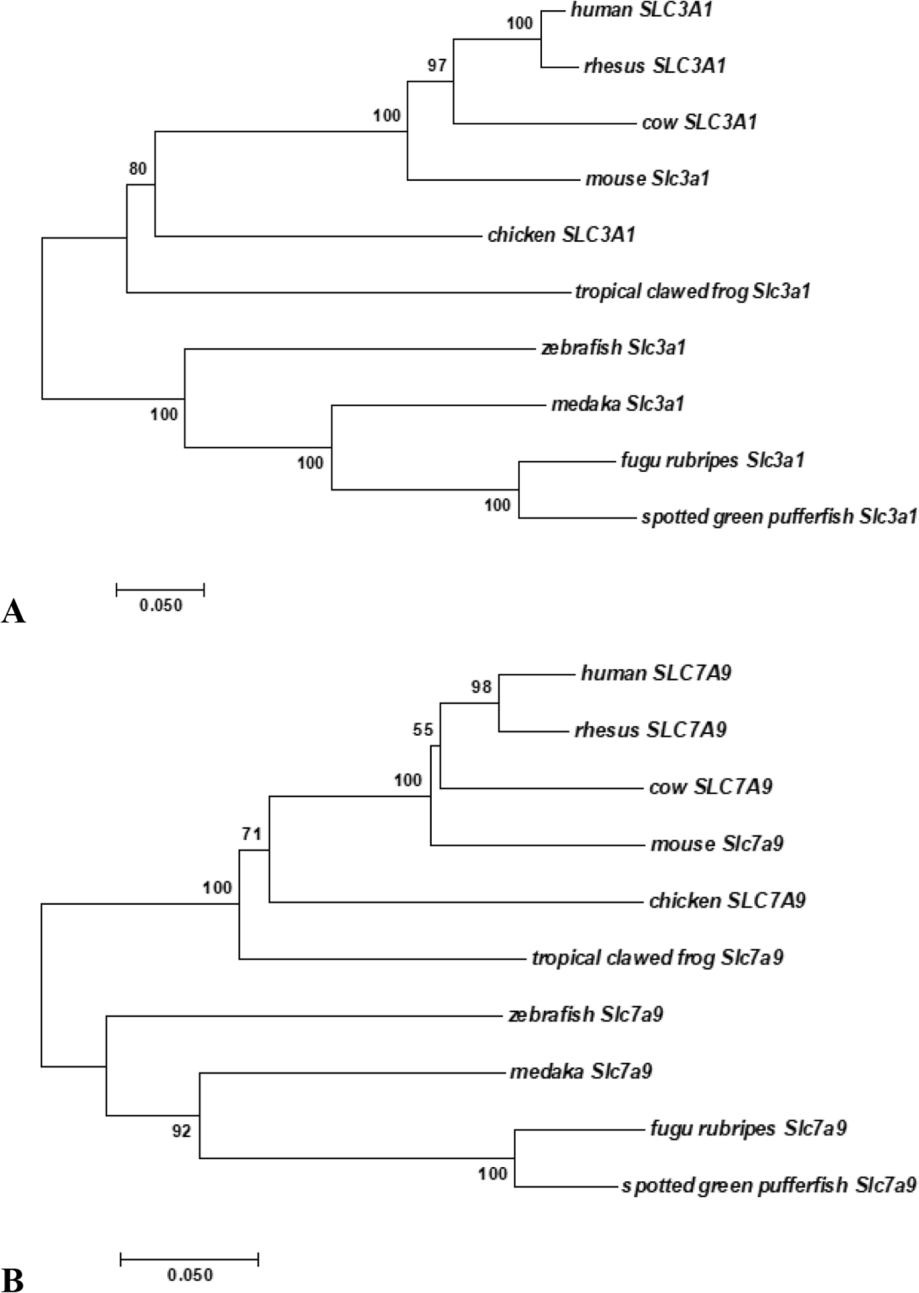
Evolutionary relationships of taxa for Slc3a1 and Slc7a9. The evolutionary history was inferred using the Neighbor-Joining method (Saitou and Nei 1987). An optimal tree with sum of branch length = 1.67110532 is shown. The percentage of replicate trees in which the associated taxa clustered together in the bootstrap test (1000 replicates) are shown next to the branches (Felsenstein 1985). The tree is drawn to scale, with branch lengths in the same units as those of the evolutionary distances used to infer the phylogenetic tree. The evolutionary distances were computed using the Poisson correction method (Zuckerkandl and Pauling 1965) and are in the units of the number of amino acid substitutions per site. The analysis involved 10 amino acid sequences. All positions containing gaps and missing data were eliminated. There were a total of 599 positions for Slc3a1 **(A)** and 471 positions for Slc7a9 **(B)** in the final dataset. Evolutionary analyses were conducted in MEGA7 (Kumar et al. 2016)

As assessed using GenBank database mining (December 2020), a single *slc3a1*-type gene was found in the zebrafish genome (GRCz11 assembly) (Table 1). Multiple alignment of Slc3a1 protein sequences from zebrafish, fugu (fugu rubripes), medaka, tetraodon (spotted green pufferfish), frog (tropical clawed frog), chicken, cow, mouse, macaca (rhesus), and human was produced using ClustalO (Sievers et al. 2011) (Fig. S1). Phylogenetic analysis showed that the teleost fish cluster was separate from that of the higher vertebrates (Fig. 2A). To evaluate whether zebrafish *slc3a1* is in conserved synteny with respect to its orthologs along the vertebrate scale, Genomicus analysis was performed. The analysis showed that *slc3a1* lays within a strong syntenic region common to zebrafish (chromosome 13), fugu rubripes (scaffold_124), medaka (chromosome 15), spotted green pufferfish (chromosome 17), tropical clawed frog (chromosome GL172661.1), chicken (chromosome 3), cow (chromosome 11), mouse (chromosome 17), rhesus (chromosome 13), and human (chromosome 2) (for further details on this specific syntenic organization see Fig. S3). Zebrafish Slc3a1 was 59–66% identical to that of the other fish species and 45–56% identical to that of higher vertebrates. As for the other vertebrates, the zebrafish Slc3a1 protein showed only one predicted transmembrane segment (amino acid 78 to amino acid 100) (Fig. S1), which consisted of 20 conserved hydrophobic amino acids flanked by glutamic acid (E) and serine (S) (conserved in all the vertebrates; Bertran et al. 1992). It was also observed that the cysteine (C) residues C^104^, C^232^, C^259^, C^565^, C^656^, C^663^, and the C-terminal C^674^ were conserved in higher vertebrates (Fig. S1). Notably, C^114^ in humans (corresponding to C^104^ in zebrafish), which is crucial for disulfide bridge formation between the light and heavy subunit chains of the heterodimer (Deora et al. 1998), was among the conserved cysteines. C^104^ in zebrafish is structurally related to this crucial disulfide bridge, as can be clearly seen in the structural representation of the zebrafish b^0,+^AT-rBAT complex in the hetero-2–2-mer form (Fig. 1).

**Table 1:** The Solute Carrier 3 family members (heavy subunits of the heteromeric amino acid transporters) in human (*Homo sapiens*) compared to zebrafish (*Danio rerio*)

Human						Zebrafish				
From: http://www.bioparadigms.org						From: http://www.ncbi.nlm.nih.gov/gene	From: https://www.ncbi.nlm.nih.gov/unigene/	From: http://zfin.org		
SLC name	Protein name	Aliases	Transport type	Substrates	Tissue and cellular expression	slc name	EST profile	Tissue and cellular expression	Stage range	References
SLC3A1	rBAT	NBAT, D2H	E (see details in Table 2)	system b^0,+^, heterodimerizes with light subunit SLC7A9	kidney, small intestine, liver, pancreas	*slc3a1 (Chr 13)*	-	this study	this study	this study
SLC3A2	4F2hc	CD98hc, FRP	E (see details in Table 2)	systems L, y^+^L, x_c_^−^ and asc with light subunits SLC7A5-8 and SLC7A10-11	ubiquitous	*slc3a2a (Chr 7) (putative slc3a2a.1)*	Developmental stage|gastrula > pharyngula ≈ adult > hatching Adult|brain > muscle ≈ kidney ≈ liver > fin > reproductive system ≈ eye	central nervous system, epiphysis, hindbrain, lens, midbrain, notochord, pancreas primordium, solid lens vesicle, telencephalon, yolk syncytial layer	2-cell to Long-pec	Rauch et al. 2003 Thisse and Thisse, 2004 Takesono et al. 2012
						*slc3a2b (Chr 21)*	Developmental stage|gastrula > larval > adult > segmentation > pharyngula > hatching Adult|brain > olfactory rosettes > gills > eye > bone ≈ heart > fin > skin > kidney ≈ reproductive system > muscle	basal plate midbrain region, cranial ganglion, diencephalon, epiphysis, enveloping layer, fin, hatching gland, hindbrain, neural tube, olfactory placode, optic tectum, periderm, peripheral olfactory organ, polster, retina, rhombomere, spinal cord, tegmentum, telencephalon, ventral mesoderm, yolk syncytial layer	Dome to Long-pec	Thisse et al. 2001 Kudoh et al. 2001 Rauch et al. 2003 Takesono et al. 2012
						*zgc:158,423*^*&*^* (Chr 7) (putative slc3a2a.2)*	Developmental stage|adult Adult|kidney > muscle	-	-	-
						*si:dkey-202g17.3 (Chr 15)*	-	-	-	-

Abbreviations for transport type: *E*: Exchanger. ^&^*slc3a2a* tandem gene

As assessed using GenBank database search (December 2020), a single *slc7a9*-type gene was found in the zebrafish genome (GRCz11 assembly) (Table 2). Multiple alignment of Slc7a9 proteins from zebrafish, fugu (fugu rubripes), medaka, tetraodon (spotted green pufferfish), frog (tropical clawed frog), chicken, cow, mouse, macaca (rhesus), and human was performed using ClustalO (Fig. S2). Phylogenetic analysis showed clustering of teleost fish Slc7a9, while Slc7a9 from the higher vertebrate branched together (Fig. 2B). To evaluate whether zebrafish *slc7a9* showed conserved synteny with respect to its orthologs along the vertebrate scale, Genomicus analysis was performed. This analysis revealed that *slc7a9* lies within a syntenic region common to zebrafish (chromosome 7), fugu rubripes (scaffold_265), medaka (chromosome 3), spotted green pufferfish (chromosome 5), tropical clawed frog (chromosome GL1722806.1), chicken (chromosome 11), cow (chromosome 18), mouse (chromosome 7), rhesus (chromosome 19), and human (chromosome 19) (for further details on this specific syntenic organization, see Fig. S4). Zebrafish Slc7a9 was 68–75% identical to that of the other fish species and 67–69% identical to that of higher vertebrates. As for the other vertebrates, zebrafish Slc7a9 exhibited 12 predicted transmembrane segments (Fig. S2). In addition, the peptide sequence alignment showed conservation of the cysteine residues C^71^, C^78^, C^88^, C^150^, C^272^, C^327^, and C^446^. A cysteine important for disulfide bridging between Slc7a9 and Slc3a1 (human C^144^, corresponding to zebrafish C^150^) was conserved in all the species investigated (Fig. S2). The zebrafish C^150^ residue is structurally related to this crucial disulfide bridge, as can be clearly seen from the structural representation of the zebrafish b^0,+^AT-rBAT complex in the hetero-2–2-mer form (Fig. 1). Alignment of human light chains revealed a 40% identity, with lower degrees of similarity toward both the N- and C-terminal ends (Wagner et al. 2001). Importantly, the C-terminal VPP (V^480^, P^481^, P^482^) from human SLC7A9, which has been shown to be responsible for endoplasmic reticulum (ER)-to-Golgi trafficking (Sakamoto et al. 2009), was found to be conserved in the zebrafish Slc7a9 C-terminal (Fig. S2). Slc7a9 also exhibits the amino acid permease domain (Pfam domain PF00324, from amino acid 41 to amino acid 473, as shown in Fig. S2), which is involved in the transport of amino acids into cells (Weber et al. 1988).

**Table 2:** The Solute Carrier 7 family members (light subunits of the heteromeric amino acid transporters) in human (*Homo sapiens*) compared to zebrafish (*Danio rerio*)

Human							Zebrafish				
From: http://www.bioparadigms.org						From: http://www.guidetopharmacology.org	From: http://www.ncbi.nlm.nih.gov/gene	From: https://www.ncbi.nlm.nih.gov/unigene/	From: http://zfin.org		
SLC name	Protein name	Aliases	Transport type	Substrates	Tissue and cellular expression	Substrates	slc name	EST profile	Tissue and cellular expression	Stage range	References
SLC7A5	LAT1	[4F2hc], 4F2lc, system L	E (similar intra- and extracellular selectivities, lower intracellular apparent affinity)	large neutral L-amino acids, T3, T4, L-DOPA, BCH	brain, ovary, testis, placenta, spleen, colon, blood–brain barrier, fetal liver, activated lymphocytes, tumor cells	large neutral amino acids including branched-chain and aromatic amino acids, miglustat	*slc7a5 (Chr 25)*	Developmental stage|pharyngula	Regenerating fin	Adult	Takayama et al. 2018
SLC7A6	y^+^LAT2	[4F2hc], system y^+^L	E (preferentially intracellular cationic amino acid against extracellular neutral amino acid/Na^+^)	cationic amino acids (Na^+^ independent), large neutral L-amino acids (Na^+^ dependent)	brain, small intestine, testis, parotids, heart, kidney, lung, thymus	L-arginine, L-lysine, L-ornithine, L-leucine, L-isoleucine, L-methionine, L-glutamine	*slc7a6 (Chr 7)*	Developmental stage|adult Adult|gills > reproductive system	(yolk) ball, yolk syncytial layer	Shield to 14–19 somites	Kudoh et al. 2001
							*si:dkeyp-120h9.1 (Chr 19)*	Developmental stage|juvenile > pharyngula > hatching > adult Adult|olfactory rosettes ≈ muscle > kidney > reproductive system	-	-	-
SLC7A7	y^+^LAT1	[4F2hc], system y^+^L	E (preferentially intracellular cationic amino acid against extracellular neutral amino acid/Na^+^)	cationic amino acids (Na^+^ independent), large neutral L-amino acids (Na^+^ dependent)	small intestine, kidney, spleen, leucocytes, placenta, lung/basolateral in epithelial cells	L-arginine, L-lysine, L-ornithine, L-leucine, L-isoleucine, L-methionine, L-glutamine	*slc7a7 (Chr 7)*	Developmental stage|adult Adult|reproductive system > kidney	brain microglial cell, myeloid cell, pancreatic D cell	26 + somites to Day 4	Rossi et al. 2015 Casano et al. 2016 Shen et al. 2016 Tarifeno-Saldivia et al. 2017
SLC7A8	LAT2	[4F2hc], system L	E (similar intra- and extracellular selectivities, lower intracellular apparent affinity)	neutral L-amino acids, T3, T4, BCH	small intestine, kidney, lung, heart, spleen, liver, brain, placenta, prostate, ovary, fetal liver, testis, skeletal muscle	most of the neutral amino acids	*slc7a8a (Chr 7)*	-	-	-	-
							*slc7a8b (Chr 2)*	-	whole organism	1-cell to Pec-fin	Thisse and Thisse, 2005
SLC7A9	b^0,+^AT	[rBAT], system b^0,+^	E (preferentially extracellular cationic amino acid and cystine against intracellular neutral amino acid)	cationic amino acids, large neutral amino acids	kidney, small intestine, liver, placenta	L-arginine, L-lysine, L-ornithine, most of the neutral amino acids	*slc7a9 (Chr 7)*	Developmental stage|adult Adult|kidney	this study	this study	this study
SLC7A10	Asc-1	[4F2hc], system asc	preferentially E	small neutral amino acids	brain, CNS, lung, small intestine, heart, placenta, skeletal muscle, kidney	L-alanine, L-serine, L-threonine, L-glutamine, glycine, D-serine	*slc7a10a (Chr 7)*	Developmental stage|adult Adult|heart > kidney > brain > eye	-	-	-
							*slc7a10b (Chr 25)*	-	-	-	-
SLC7A11	xCT	[4F2hc], system x_c_^−^	E (preferentially extracellular cystine against intracellular glutamate)	cystine (anionic form), L-glutamate	macrophages, brain, retinal pigment cells, liver, kidney	L-cystine, L-glutamic acid	*slc7a11 (Chr 14)*	Developmental stage|adult Adult|kidney	-	-	-
							*si:ch73-352p4.8 (Chr 4)*	Developmental stage|hatching > pharyngula Adult|muscle	-	-	-
SLC7A13	AGT-1	XAT2	E	L-aspartate, L-glutamate	proximal straight tubules, distal convoluted tubules	-	-	-	-	-	-
SLC7A14			O		highly expressed in CNS	-	*slc7a14a (Chr 2)*	Developmental stage|hatching > adult Adult|brain > eye	-	-	-
							*slc7a14b (Chr 24)*	-	-	-	-
SLC7A15P^&^	Pseudogene	-	-	-	-	-	-	-	-	-	-
-	-	-	-	-	-	-	*zmp:0,000,001,267 (Chr 17)*	-	-	-	-

Abbreviations for transport type: *C*, cotransporter; *E*, exchanger; *F*, facilitator transporter; *O*, orphan transporter. ^&^Mouse *Slc7a15* is functional. *BCH*, 2-amino-2-norbornanecarboxylic acid; *CNS*, central nervous system; *T3*, triiodothyronine; *T4*, thyroxine; *L-DOPA*, precursor to catecholamine neurotransmitters

### Expression of *slc3a1* and *slc7a9* during zebrafish nephron development

**Fig. 3:**
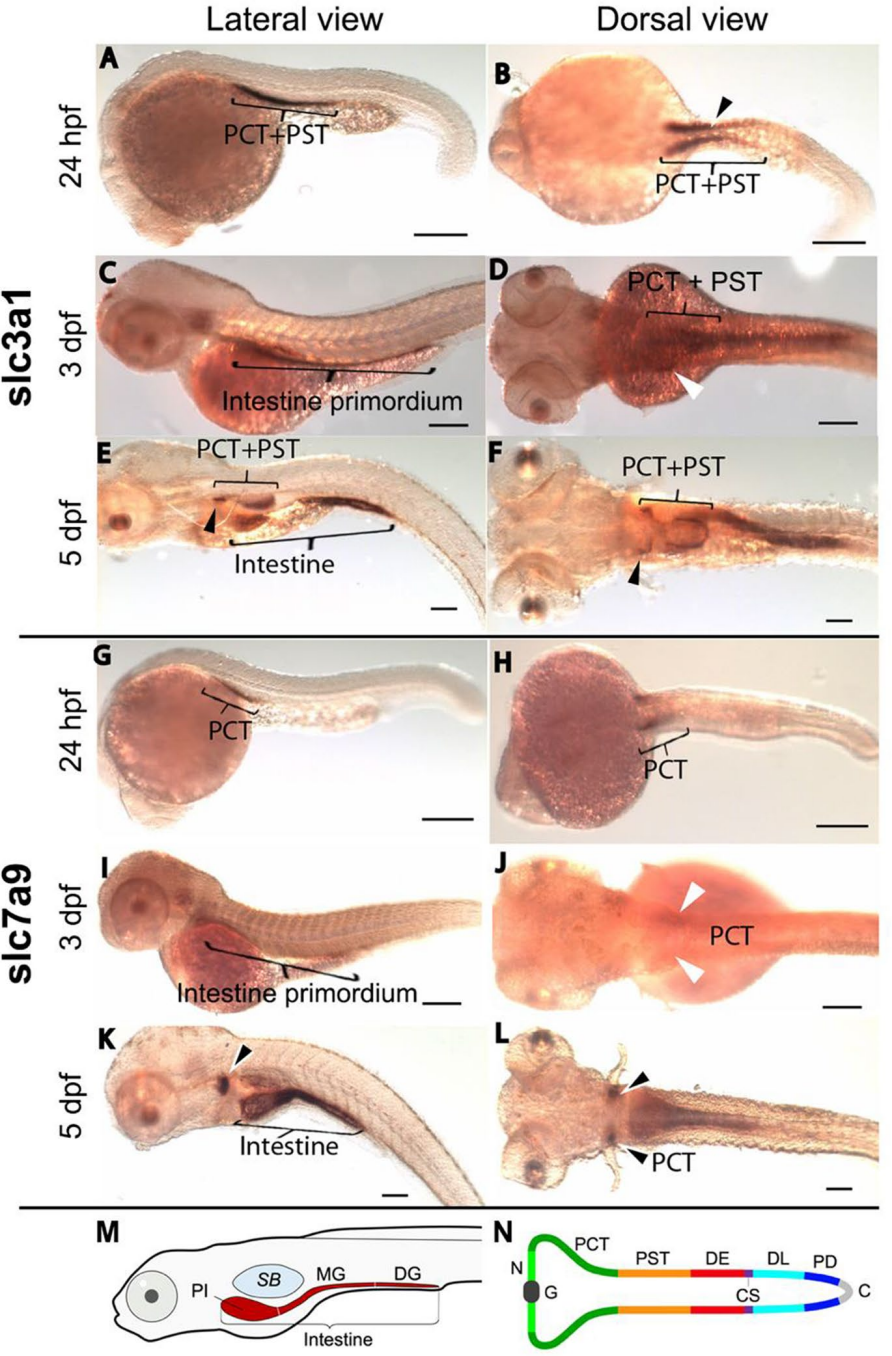
Spatiotemporal distribution of *rBAT/slc3a1* and *b*^*0,*+^*AT/slc7a9*. Whole-mount in situ hybridization of wild-type embryos. **(A)** Lateral view shows expression of *slc3a1* in proximal convoluted tubule (PCT) and proximal straight tubule (PST) segments of the nephron at 24 hpf. **(B)** Dorsal view shows two parallel stripes of *slc3a1* expression at 24 hpf in the pronephros-spanning regions of PCT and PST. **(C)** Lateral view shows *slc3a1* expression in intestinal primordium at 3 dpf. **(D)** *slc3a1* expression at 3 dpf is also observed in the PCT and PST in the dorsal view. **(E**–**F)** *slc3a1* expression in the intestine, PCT, and PST at 5 dpf. **(G)** Lateral view and **(H)** dorsal view show the *slc7a9* expression only in the PCT at 24 hpf. At 3 dpf, *slc7a9* expression is seen in the intestinal primordium **(I)** and PCT **(J)**. At 5 dpf, *slc7a9* expression is seen in the PCT **(K-L)** and intestine **(K–L)**. Arrowhead in **B** indicates putative region between PCT and PST. Arrowheads in **D, E, F, J, K,** and **L** indicate PCT expression domains (scale bar: 100 µm). **M** Schematic lateral view of primordial intestine at 5 dpf; with proximal intestine (PI), midgut (MG) and distal gut (DG). SB, swim bladder. **N** Schematic dorsal view of nephrons with segments indicated: glomerolus (G), neck (N), distal early (DE), corpuscle of Stannius (CS), distal late (DL), and pronephric duct (PD), cloaca (C) (modified from Wingert et al. 2007). Abbreviations: hpf, hours post fertilization; dpf, days post fertilization

We investigated the spatiotemporal distribution of *slc3a1* and *slc7a9* transcripts in the nephron and digestive system of wild-type zebrafish embryos using whole-mount in situ hybridization between 24 h post fertilization (hpf) and 5 dpf. Recent terminology for zebrafish nephron segmentation was adopted from Wingert and colleagues (Wingert et al. 2007). The first two zebrafish nephrons arise from the intermediate mesoderm and form eight distinct segments (going from proximal to distal): glomeruli, neck, proximal convoluted tubule (PCT), proximal straight tubule (PST), distal early, corpuscle of Stannius, distal late, and pronephric duct (Fig. 3M, N) (Wingert et al. 2007). This differentiation of segments helped us ascertain the specific nephron segments for HATs according to their expression patterns. At 24 hpf, both PCT and PST segments expressed *slc3a1* (Fig. 3A)*,* while *slc7a9* was expressed only in the PCT (Fig. 3G). *slc7a9* expression is similar to that of another solute carrier, *slc20a1a,* involved in Na^+^/PO_4_ cotransport, which is also expressed in the PCT segment at 26 hpf (Wingert et al. 2007). For both *slc3a1* and *slc7a9*, two distinct, paraxial expression domains (parallel to the midline) in the proximal segments of the pronephros were observed at 24 hpf (Fig. 3B,H). The distinction between the PCT and PST at 24 hpf was marked by shorter and more posteriorly restricted expression of *slc7a9* limited to the PCT (Fig. 3G), as compared to that of *slc3a1*, which extended more posteriorly and spanned both the PCT and PST regions (Fig. 3A). The occurrence of HATs at this early stage and in specific segments marks the maturation and differentiation of segment-specific epithelia before the onset of renal blood filtration, which occurs at 40 hpf (Drummond 2000, 2003, 2004, 2005). PCT coiling was observed between 72 and 144 hpf (Wingert et al. 2007); at 3 dpf, this was marked by the weak expression of *slc3a1* (Fig. 3D) and *slc7a9* (Fig. 3J). PCT coiling and positioning anterior to the PST was evident at 5 dpf in the dorsal view, where *slc3a1* was expressed in the PCT plus PST (Fig. 3F), while *slc7a9* was restricted to the PCT (Fig. 3L). The segment identities of PCT and PST were assigned using the expression patterns previously determined for *slc20a1a* (in PCT) and for *trpm7* and *slc13a1* (in PST) at 26 hpf and 48–144 hpf, respectively (Wingert et al. 2007).

### Expression of *slc3a1* and *slc7a9* during zebrafish intestine development

Although the zebrafish intestinal lumen starts forming at 26–52 hpf (Ng et al. 2005), the expression of HATs in the intestinal primordium was observed much later, at 3 dpf (Fig. 3C,I). Expression of *slc3a1* (Fig. 3C) and *slc7a9* (Fig. 3I) is marked by a line arising anteriorly from the pectoral fin bud posteriorly till the cloaca. By 74–76 hpf, the entire digestive tract is a hollow tube formed by epithelial cell polarization (Ng et al. 2005). At 5 dpf, the remodeling and differentiation of zebrafish intestinal epithelium takes place, the intestinal tract is segmented into the intestinal bulb, mid intestine, and posterior intestine (Ng et al. 2005), and the yolk is completely resorbed (Fig. 3E,K). At this stage, the larva is ready for exogenous feeding, which means it should start absorbing nutrients through the functional intestinal epithelium. Increased co-expression of *slc3a1* (Fig. 3E) and *slc7a9* (Fig. 3K) along the entire intestine marks this transition in nutrient supply.

### *slc7a9* and *slc3a1* are expressed in kidney and intestine-polarized cells (nephron tubule cells and enterocytes)

**Fig. 4:**
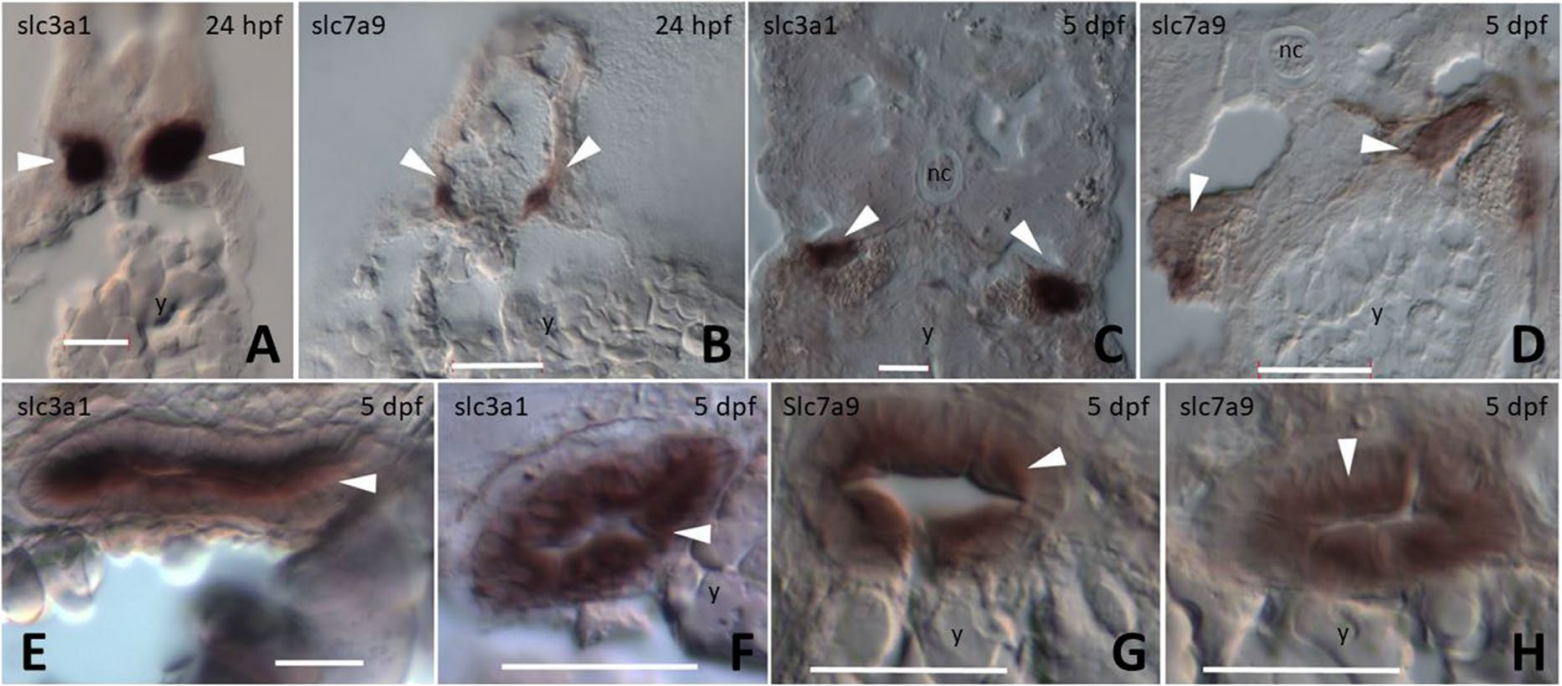
Images of cryo cross-sections from whole-mount in situ hybridization for the genes *slc3a1* and *slc7a9*. **(A)** *slc3a1* and **(B)** *slc7a9* expression in two distinct nephrons (white arrowheads) at 24 hpf. **(C)** *slc3a1* and **(D)** *slc7a9* expression in two distinct nephrons (white arrowheads) at 5 dpf. **(E–F)** *slc3a1* and **(G-H)** *slc7a9* intestinal cross-sections reveal the localization of expression in enterocytes at 5 dpf (white arrowheads). Scale bar: 20 µm. All sections were dorsal to the top and ventral to the bottom. Scale bar: 20 µm. Abbreviations: nc, notochord; y, yolk; hpf, hours post fertilization; dpf, days post fertilization

To focus on the detailed position of *slc3a1* and *slc7a9* expression in the nephron and the structural organization of the epithelial cell lining of the intestine, we generated serial transverse sections. Cross-sections of the whole-mount in situ hybridized embryos revealed strong expression of *slc3a1* along the anterior–posterior length of the nephron, ventral to the notochord and somites, and dorsal to the yolk at 24 hpf (Fig. 4A). A similar position but weaker expression of *slc7a9* was observed in the cross-section from the most anterior part of the nephron at 24 hpf (Fig. 4B). *slc3a1* was expressed in the PCT and PST segments of the pronephric ducts running ventral and parallel to the somite muscles at 5 dpf (Fig. 4C). *slc7a9* expression was similar to that of *slc3a1*, but restricted to sections from the PCT region of the anterior nephrons (Fig. 4D). Co-expression of *slc3a1* and *slc7a9* in the PCT (Figs. 3, and 4) supports that *slc7a9* and *slc3a1* act together in the PCT region of zebrafish. At 3 dpf, both *slc3a1* and *slc7a9* showed similar expression domains in the intestine (Fig. 3C,I). Interestingly, the intestinal cryo cross-sections from 5 dpf embryos revealed the localization of *slc3a1* mRNAs to the luminal single layer of enterocytes (Fig. 4E–F, white arrowhead). This epithelial cellular mRNA localization was also clearly visible for *slc7a9* at 5 dpf (Fig. 4G–H, white arrowhead).

## Discussion

Absorption of amino acids from the intestine and their reabsorption in the kidney to prevent urinary loss is highly relevant for protein synthesis, accretion, and body growth. This process is vital in case of essential amino acids, such as lysine. Notably, impairment of renal amino acid reabsorption leads to various types of aminoacidurias. In this study, we focused on the system b^0,+^, which is involved in the transport of lysine, arginine, ornithine, and cystine. Our sequence comparison identified the zebrafish system b^0,+^ along with information on its syntenic conservation with functionally- and developmentally-related genes. Analysis of gene expression patterns at various stages revealed the nephron segment-specific and intestinal occurrence of both *slc3a1* and *slc7a9.* Further analysis using transverse sections revealed system b^0,+^ transcript localization in polarized intestinal epithelial cells.

### System b^0,+^ protein sequences are evolutionarily conserved

System b^0,+^ is involved in crucial functions of essential amino acid transport and is highly conserved from invertebrates to mammals. HATs have been shown to be functionally conserved throughout evolution from nematodes to mammals (Veljkovic et al. 2004). The membrane topology predictions for zebrafish *slc3a1* and *slc7a9* showed one and twelve transmembrane domains, respectively, which is in agreement with previous reports (Wagner et al. 2001). In addition, the cysteine residues involved in the formation of the disulfide bridge in HATs from higher vertebrates are conserved in the zebrafish system b^0,+^. Also, the C-terminus of b^0+^AT shows that the VPP motif (V^480^, P^481^, P^482^) is conserved in zebrafish. This motif is reported to be responsible for trafficking of the heterodimer SLC3A1/SLC7A9 from the ER to the Golgi apparatus (Ganapathy 2009; Sakamoto et al. 2009). It is still unknown whether the VPP/VVY C-terminal sequences in other light chains play a role in ER-Golgi trafficking of the respective heterodimers (Ganapathy 2009; Sakamoto et al. 2009) or if it exerts specificity of the light chain towards a particular heavy chain. Because of the conservation of the C-termini of light chains, common regulatory mechanisms involving C-termini action among HATs might be present, as proposed (Sakamoto et al. 2009).

On the other hand, the cysteine residue located at the C-terminal (C^674^) of zebrafish Slc3a1 is fully conserved in higher vertebrates. This is in line with the observation that deletion of the human SLC3A1 C-terminal disulfide loop (residues 673–685) prevents maturation and prompts degradation of the transporter (Rius et al. 2016). Taken together, similar membrane topology and conservation of functionally vital residues and motifs confirmed the identities of the predicted zebrafish system b^0,+^ orthologs.

### *slc3a1* and *slc7a9* occur in a non-duplicated form in the zebrafish genome

Single *slc3a1*- and *slc7a9*-type genes are present in the zebrafish genome. This was primarily concluded from the initial GenBank database mining and was subsequently confirmed by thorough and systematic consulting of other platforms and databases such as Ensembl and UniProt. As part of our strategy, we searched for genes/proteins and used the alignment tools associated (e.g., BLAST) to cross-check the sequence analysis. In addition, whenever possible (e.g., in case of BLAST analysis at NCBI), not only the non-redundant or RefSeq Select or Reference sequence sections, but also more explorative sections, such as the Transcriptome Shotgun Assembly or the Expressed sequence tags sections, were systematically consulted. In this way, we thoroughly investigated the large family of sequences present in the various databases related to Slc3a1 and Slc7a9 in zebrafish, in order to fully assess the effective number of *slc3a1*- and *slc7a9*-type genes and define the related Slc3a1- and Slc7a9-type proteins.

### System b^0,+^ is expressed in developing zebrafish kidney and intestine

We found that the system b^0+^, which is involved in lysine, arginine, ornithine, and cystine transport, in addition to that of (large) neutral amino acids, is expressed in the developing nephron (24 hpf) much earlier than in the intestine (3 dpf). This indicates that the b^0+^-mediated renal reabsorption of amino acids is activated earlier than intestinal absorption. This is possibly related to the close homeostatic control of the amino acid pool in the developing embryo, which includes recovery of important solutes from the pre-urine. Exogenous feeding starts later (after 5 dpf), and prior to this, the zebrafish acquire amino acids and other nutrients from the yolk as a sole source.

Both *slc3a1* and *slc7a9* show high levels of expression in the proximal tubule part of pronephros; however, differences in spatial distribution were detectable, with *slc7a9* expression restricted to the PCT, while *slc3a1* showed stronger expression in the PCT and PST. These differences in the pronephric distribution of expression in zebrafish are consistent with those reported in mice (Palacin et al. 2001) and *Xenopus* (Raciti et al. 2008). The overlap of *slc3a1*-*slc7a9* expression in the proximal tubule segments is in correlation with this heterodimer, which is responsible mainly for cystine transport from the kidney proximal tubule, as discussed by Fernandez et al. (2002). Since the light chain, i.e., *slc7a9*, is only present in the PCT region, whereas the heavy chain *slc3a1* expression extends to the PST segment (Furriols et al. 1993), alternative light chain partners for *slc3a1* and heavy chain partners for *slc7a9* are likely to exist in the non-overlapping expression domains (Palacin et al. 2001). Preliminary data from morpholino knock-down experiments targeting *slc7a9* might indicate that *slc7a9* is important for proper formation of the PCT segment were morphants lacked the convolution of the proximal segment of the nephron (Supplementary Materials II). However, these results should be followed up with generation and analysis of a stable *slc7a9* mutant zebrafish line (e.g., by utilizing Cas9-CRISPR technology).

A gradual increase in the intestinal co-expression of *slc3a1* and *slc7a9* from 3 to 5 dpf is in coordination with the functional maturity of the zebrafish intestine (Ng et al. 2005; Verri et al. 2003). A stronger expression at 5 dpf might be part of the preparations of the transport machinery for the onset of exogenous feeding since most of the yolk has been resorbed by this time.

This is the first report on the system b^0,+^ being expressed in the developing kidney and intestine in zebrafish as early as 24 hpf, 3 dpf, and 5 dpf.

## Supplementary Information

Below is the link to the electronic supplementary material.

Supplementary file1 (DOC 426 KB)

Supplementary file2 (DOCX 615 KB)

## Data Availability

All the data is available in the paper and the supplementary material (Supplementary Materials I and Supplementary Materials II).
