# Supplementary material for: The zebrafish cationic amino acid transporter/glycoprotein-associated family: sequence and spatiotemporal distribution during development of the transport system b0,+ (slc3a1/slc7a9)
Source: Fish Physiol Biochem. 2021 Aug 2;47(5):1507–25. doi: 10.1007/s10695-021-00984-z (PMC8478756; doi:10.1007/s10695-021-00984-z)
Supplement: Supplementary file 1 — Supplementary file1 (DOC 426 KB) [file 10695_2021_984_MOESM1_ESM.doc]

**The zebrafish cationic amino acid transporter/glycoprotein-associated family: sequence and spatiotemporal distribution during development of the transport system b^0,+^ (*slc3a1*/*slc7a9*)**

Ståle Ellingsen, Shailesh Narawane, Anders Fjose, Tiziano Verri, Ivar Rønnestad

**Supplementary Materials I**

**Table S1**

**Table S2**

**Fig. S1**

**Fig. S2**

**Fig. S3**

**Fig. S4**

**Appendix I**

**Table S1.** Ensembl peptide IDs for *slc3a1*- and *slc7a9*-related sequence analysis and comparison.

| **Species** | **Gene name** | **Ensembl peptide ID** | **UniProtKB peptide ID** | **Gene name** | **Ensembl peptide ID** | **UniProtKB peptide ID** |
| --- | --- | --- | --- | --- | --- | --- |
| Human | *SLC3A1* | ENSP00000260649 | Q07837 | *SLC7A9* | ENSP00000023064 | P82251 |
| Macaque | *SLC3A1* | ENSMMUP00000008308 | F7HIT7 | *SLC7A9* | ENSMMUP00000031688 | F6XDS9 |
| Mouse | *Slc3a1* | ENSMUSP00000024944 | Q91WV7 | *Slc7a9* | ENSMUSP00000032703 | Q9QXA6 |
| Cow | *SLC3A1* | ENSBTAP00000023161 | Q3SZF7 | *SLC7A9* | ENSBTAP00000034433 | Q3ZCL6 |
| Chicken | *SLC3A1* | ENSGALP00000016193 | E1C011^&^ | *SLC7A9* | ENSGALP00000007521 | E1BT87^$^ |
| Tropical clawed frog | *slc3a1* | ENSXETP00000005991 | Q0V9A1 | *slc7a9* | ENSXETP00000039600 | F7C436 |
| Zebrafish | *slc3a1* | ENSDARP00000114538 | F1QEA9 | *slc7a9* | ENSDARP00000091252 | F1QGJ2 |
| Medaka | *slc3a1* | ENSORLP00000005633 | H2LI15 | *slc7a9* | ENSORLP00000014785 | H2M8G9 |
| Fugu rubripes | *slc3a1* | ENSTRUP00000019541 | H2T4F3 | *slc7a9* | ENSTRUP00000000655 | H2RKJ2^£^ |
| Spotted green pufferfish | *slc3a1* | ENSTNIP00000010276 | H3CPU3 | *slc7a9* | ENSTNIP00000007317 | H3CGD9 |

^&^see also GenBank Acc. No. XP_004935427.1

^$^see also GenBank Acc. No. NP_001186062.1

^£^see also GenBank Acc. No. XP_011616887.1

^§^see also Ensembl peptide ID ENSTNIP00000000982 (UniProtKB peptide ID H3BYB6)

**Table S2.** Primers used for *in situ* probe preparation.

| **Gene symbol** | **Forward primer 5**'**🡪3**' | **Reverse primer 5**'**🡪3**' |
| --- | --- | --- |
| *slc3a1* | ACAGCCAGATCTGAACTATCGTAAC% | ATGGAGTGTGAGAGAATCTGGTAAC^‡^ |
| *slc7a9* | CATCTGTCTGATAGTGGGTACAATG%% | TTAAACATTAGAGCTGGAGAAGGTG^‡‡^ |

%*slc3a1*: in coding exon 4

^‡^*slc3a1*: in coding exon 10

%%*slc7a9*: in coding exon 2

^‡‡^*slc7a9*: in coding exon 9

zebrafish_Slc3a1 MS-LTRVTNIDAVELQEGIQNAAFREDDDD---------------NNGDIVCIRRVSSLP 44

medaka_Slc3a1 MS-YIRKD-SGSVELADGIRNLGFQDADVV---TDFPPH-----SSPRDAVERKDSAAEI 50

fugu_rubripes_Slc3a1 ------IW-NAVMALVRYHTNPGFEDVEVGETTAELNTV-----PEEKGC-SAAGTRDVD 47

spotted_green_pufferfish_Slc3a1 ----------------------------------ELPTD-----PRERASVAGPRAEEED 21

tropical_clawed_frog_Slc3a1 ---MEEDPNSVELKQKAGHENNGFISDHDGKEEGKPEKVEIRRNSKEEDTHIYSVRI-DE 56

chicken_SLC3A1 MVDKELKGLPMELSEKGGVENNGFVQNEAFDDRDTDTSS------QDEIPKTCAVDVS-P 53

mouse_Slc3a1 MDEDKGKRDPIQMSLKGCRTNNGFVQNEDIPEQDPDPGS------R-DTPQPNAVSIPAP 53

cow_SLC3A1 MAEDKSKRDSIGLNAKEGQTNNGFVQNEDIRETDLDPSS------PVVGPQHNTVDILGP 54

human_SLC3A1 MAEDKSKRDSIEMSMKGCQTNNGFVHNEDILEQTPDPGS------STDNLKHSTRGILGS 54

rhesus_SLC3A1 MAEDKSKRDSIGMNMKGCQTNNGFVHNEDILEQTPDTGS------SADNLQHSTTSILGS 54

**<----------I----------> ↓**

zebrafish_Slc3a1 EESEYTQIKPYAGMPKEVLMLYSNKPCYRIPREVIFWLVIACTLALIAMTAAIVALSPRC 104

medaka_Slc3a1 DDSVYAQVEPYAGMPKEVLLLYSSQARYRVPREVLFWLTVCCTLALVAVTVAIIALSPPC 110

fugu_rubripes_Slc3a1 ASEEYTQIKPYPGMPKEVLLLYSSQARYRVPREIIFWLTVACTAALVGLTATVIALSPRC 107

spotted_green_pufferfish_Slc3a1 PSAEYAQIKPYAGMPREVLLLYSSQARYRVPREIIFWLTVACTAALVGLTVTVIVLSPRC 81

tropical_clawed_frog_Slc3a1 IPENAPPLKPYAGMPKDVLLQFSNQPCYRIAREIIFWLIIVATLAIIAATIAIIAISPKC 116

chicken_SLC3A1 AAVAEPALQPYAGMPKEVLLKFSSQARYRVTREILFWLTIAAAVLLVSATIAIIALSPKC 113

mouse_Slc3a1 EEPHLKAVRPYAGMPKEVLFQFSGQARYRVPREILFWLTVVSVFLLIGATIAIIVISPKC 113

cow_SLC3A1 GEPDVKDVRPYAGMPKEVLFQFSGQACYRIPREVLFWLTVASVLVLIAATIAIIAISPKC 114

human_SLC3A1 QEPDFKGVQPYAGMPKEVLFQFSGQARYRIPREILFWLTVASVLVLIAATIAIIALSPKC 114

rhesus_SLC3A1 QEPDFKGVQPYAGMPKEVLFQFSGQARYRIPREILFWLAVASVLVLIAATVAIIVLSPKC 114

:.** ***::**: :*.: **: **::*** : .. ::. * :::.:** *

zebrafish_Slc3a1 MSWWQLSPIYQVYPRSFKDSNADGVGDLRGIKEKLSHFEYLNIKAIWISPFYKSPMRDFG 164

medaka_Slc3a1 LGWWQISPVYQVYPRSFKDSDGDGVGDLRGIKEKLDHFHSLNIKSIWISPFYRSPMKDFG 170

fugu_rubripes_Slc3a1 LSWWQTSPMYQLYPRSFKDSDGDGIGDLKGILQKLDHFQYLNIKSVWIGPLYRSPMKDFG 167

spotted_green_pufferfish_Slc3a1 LSWWQTSPVYQLYPRSFKDSDGDGVGDLKGILEKLDHFQYLNIKSVWIGPLYRSPMRDFG 141

tropical_clawed_frog_Slc3a1 LDWWQRSPIYQVYPKSFKDSNNDGSGDLKGVQEKIDHFIYLDVKNIWVAPFYKSSLKDFN 176

chicken_SLC3A1 LDWWQDGPIYQVYPRSFKDSNMDGNGDLKGIQEKLDHITHLNIKTIWITSFYKSPLKDAG 173

mouse_Slc3a1 LDWWQAGPIYQIYPRSFKDSDKDGNGDLKGIQEKLDYITALNIKTLWITSFYKSSLKDFR 173

cow_SLC3A1 LDWWQAGPMYQIYPRSFRDSNKDGDGDLKGIQDKLDYITTLNIKTVWITSFYKSSLKDFR 174

human_SLC3A1 LDWWQEGPMYQIYPRSFKDSNKDGNGDLKGIQDKLDYITALNIKTVWITSFYKSSLKDFR 174

rhesus_SLC3A1 LDWWQEGPMYQIYPRSFKDSNKDGNGDLKGIQDKLDYITALNVKTVWITSFYKSSLKDFR 174

:.*** .*:**:**:**:**: ** ***:*: :*:.:: *::* :*: :*:* ::*

zebrafish_Slc3a1 YDVEDFRDVDPLFGTMEDFDDLLTSMHDKGLKLIMDYIPNHTSDKHVWFQLSRNYTEPYT 224

medaka_Slc3a1 YDVEDFRDVDPLFGTMEDFEELLAEMHKNGLKLIMDFIPNHSSDRHRWFNLSRTRDPLYE 230

fugu_rubripes_Slc3a1 YDVEDFRSISPEFGTMQDFEELLAEMHNRGLNLIMDFIPNHTSDRHPWFILSRTRDPQYE 227

spotted_green_pufferfish_Slc3a1 YDVEDFRSISPEFGTMQDFEELLAEMHNRGLNLIMDFIPNHTSDRHPWFNLSRTGDPHYR 201

tropical_clawed_frog_Slc3a1 YAVDDHMEVDPTFGTMADFDSMISAMHDKGLKLIIDLIPNHTSNKHKWFQLSRNRTDKYT 236

chicken_SLC3A1 YGAEDFYDIDPMFGSMRDFEDLLASIHDRGLKVIMDFIPNHTSDKHQWFQLSRNRTGKYT 233

mouse_Slc3a1 YAVEDFKEIDPIFGTMKDFENLVAAIHDKGLKLIIDFIPNHTSDKHPWFQSSRTRSGKYT 233

cow_SLC3A1 HGVEDFREIDPIFGTMKDFENLVAAIHDKGLKLIIDFIPNHTSDKHAWFQWSRNQTGKYT 234

human_SLC3A1 YGVEDFREVDPIFGTMEDFENLVAAIHDKGLKLIIDFIPNHTSDKHIWFQLSRTRTGKYT 234

rhesus_SLC3A1 YGVEDFRQVDSIFGTMEDFENLVAAIHDKGLKLIIDFIPNHTSDKHTWFQLSRTRTGKYT 234

: .:*. .:. **:* **:.::: :*..**::*:* ****:*::* ** **. *

zebrafish_Slc3a1 DYYIWVNCTAD----KHPNNWVSVFGNSTWEYDEIRQQCYFHQFLKEQPDLNYRNPLVLQ 280

medaka_Slc3a1 DYYVWADCNAT----KKPNNWVSIFGNSSWTYDEVRGQCYLHQFLKEQPDLNMRNPAVRK 286

fugu_rubripes_Slc3a1 DYYIWADCNQT---APRPNNWVSVFGNTSWAYDEVRGQCYLHQFFKEEPDLNLRNPRVRK 284

spotted_green_pufferfish_Slc3a1 DYYVWADCNQT---AARPNNWVSVFGNSSWTYDDVRGQCYLHQFFKEQPDLNLRNPSVRR 258

tropical_clawed_frog_Slc3a1 DYYIWHDCAQVGGVRVPPNNWVSVYGDSAWEYDVTRNQCYLHQFRKEQPDLNFNNPDVHE 296

chicken_SLC3A1 DYYIWQDCVQAGAAISAPNNWVSVYGNSSWQYDDVRKQCYFHQFGKEQPDLNFRNPAVQQ 293

mouse_Slc3a1 DYYIWHNCTHVNGVTTPPNNWLSVYGNSSWHFDEVRKQCYFHQFLKEQPDLNFRNPAVQE 293

cow_SLC3A1 DYYIWHDCNRENGTTIPPNNWLSVYGNSSWHFDEVRKQCYFHQFMKEQPDLNFRNPDVQE 294

human_SLC3A1 DYYIWHDCTHENGKTIPPNNWLSVYGNSSWHFDEVRNQCYFHQFMKEQPDLNFRNPDVQE 294

rhesus_SLC3A1 DYYIWHDCTHENGTTVPPNNWLSVYGNSSWHFDEVRNQCYFHQFMKEQPDLNFRNPDVQE 294

***:* :* ****:*::*:::* :* * ***:*** **:**** .** * .

zebrafish_Slc3a1 EMTDIIHFWLKKGVDGFRMDAVKHMLEATHLRDEPQVNPDQDPSTVDTEFELYHDYTYTQ 340

medaka_Slc3a1 EIIDIIHFWLSKGVDGFRMDAVKHMLEATHLRDEPQVDPNKPPEEVTTEWDLFHDYTTSQ 346

fugu_rubripes_Slc3a1 EMIDIIRFWLEKGVDGFRIGSAKYALEAAHLRDEPQVDPNKPAESVTSDEDLHRDYTTSQ 342

spotted_green_pufferfish_Slc3a1 EMIDIIRFWLEKGVDGFRIGSVKYVLEAAHLRDEPQVDPKKPAESVTADKDLHRDYTTSQ 318

tropical_clawed_frog_Slc3a1 EILNIIKFWLGKGVDGFTINSAKFILEAEHFRDEPQVNKLQDPATISNYAELFHDYTTTQ 356

chicken_SLC3A1 EIHDVIKFWLGKGVDGFNFIAVKFLLEATHLRDEPQVNKSQNPESITAYSELYHDYTTTQ 353

mouse_Slc3a1 EIKEIITFWLSKGVDGFSFDAVKFLLEAKDLRNEIQVNTSQIPDTVTHYSELYHDFTTTQ 353

cow_SLC3A1 EIKEIIQFWLSKGVDGFSFDALPFLLEAKHLRDEAQVNKTQIPDMVTHYSQLHHDFTTTQ 354

human_SLC3A1 EIKEILRFWLTKGVDGFSLDAVKFLLEAKHLRDEIQVNKTQIPDTVTQYSELYHDFTTTQ 354

rhesus_SLC3A1 EIKEILQFWLTKGVDGFSFDAVKFLLEAKHLRDEIQVNKTQIPNTVTQYSELYHDFTTTQ 354

*: ::: *** ****** : : . *** .:*:* **: : : :*.:*:* :*

zebrafish_Slc3a1 AGLHEILTDWRIQMDTYSREPGRYRFMVMESYDYEEIDKTMRYYGTNYAKESDFPFNFYL 400

medaka_Slc3a1 VGLHDILREFRAEMEPYSREPGKYRFMVTESYDYEEVEKTMMYYGTPLVKESDFPFNFYL 406

fugu_rubripes_Slc3a1 LGLHDLLREWRAEMDAYSHEPGRYRLMVTESYDYHEVEKTMMYYSTPLAKESDLPFNFYL 402

spotted_green_pufferfish_Slc3a1 LGLHDLLREWRAEMDAYSREPGRYRLMVTESYDDHEVEKTMMYYSSPLVKESDFPFNFYL 378

tropical_clawed_frog_Slc3a1 VGMHDIIRNFRQTINKYSREPGRYRFMGTESNDQPAIDKTMLYYGNSFIQEADFPLNSYL 416

chicken_SLC3A1 VGMHDIIRSFRQTMDQYSSEPGRYRFMGSDSDEKEDIEATMMYYGTTFVQEADFPFNLHL 413

mouse_Slc3a1 VGMHDIVRDFRQTMNQYSREPGRYRFMGAEAS-AESIERTMMYYGLPFIQEADFPFNKYF 412

cow_SLC3A1 VGMHDIVRSFRQTMNQYSREPGRYRFMGTEAH-GESITKTMVYYGLPFIQEADFPFNSYL 413

human_SLC3A1 VGMHDIVRSFRQTMDQYSTEPGRYRFMGTEAY-AESIDRTVMYYGLPFIQEADFPFNNYL 413

rhesus_SLC3A1 VGMHDIVRSFRQTMDQYSTEPGRYRFMGTEAY-AETIDRTVMYYGLPFIQEADFPFNNYL 413

*:*::: .:* :: ** ***:**:* :: : *: **. :*:*:*:* ::

zebrafish_Slc3a1 LDLP-DNLSGNYAKSIVERWMSNMPKGKWPNWVVGNHDKPRIGSSAGKEYVRALNMLLLT 459

medaka_Slc3a1 LDLP-QNASGLWAKHLVDLWMSNMPRGKWPNWVVGNHDRSRIASSAGKMYVRVINMLLLT 465

fugu_rubripes_Slc3a1 LDLP-QNNSGLWVKQLVDLWMANMPRGQWANWVVGNHDKPRIASTAGQTYVALINMLLLT 461

spotted_green_pufferfish_Slc3a1 LDLP-QNSSGLWARQLVHLWMASMPRGQWANWLVGNHDKPRIASAAGQTYVALFNMLLLT 437

tropical_clawed_frog_Slc3a1 LDLSRTNLNGLSIFSNVDLWMKSMPSGKWPSWMVGGPSYSRISSRVGRQYINVMNMLLLT 476

chicken_SLC3A1 INMK--NLSGNSVFEAVNMWMKNMPEGKWPNWAVGSPNTARISSRFGSEYVRVINMLLLT 471

mouse_Slc3a1 TTIG--TLSGHTVYEVITSWMENMPEGKWPNWMTGGPETPRLTSRVGSEYVNAMHMLLFT 470

cow_SLC3A1 SKLD--KPSGNSVSEIITSWMENMPEGKWPNWMTGGPDSVRLTSRLGEKYVNIMNMLVFT 471

human_SLC3A1 SMLD--TVSGNSVYEVITSWMENMPEGKWPNWMIGGPDSSRLTSRLGNQYVNVMNMLLFT 471

rhesus_SLC3A1 TMLD--TVSGNSVYEVITSWMENMPEGKWPNWMIGGPDSSRLTSRLGNEYVNVMNMLLFT 471

: . .* : ** .** *:* .* *. . *: * * *: ::**::*

zebrafish_Slc3a1 LPGTPTTYYGEEIGMVDVNISV--IQDPAGQYDPSKSRDPQRTPMQWNNELNAGFSESLN 517

medaka_Slc3a1 LPGTPTTYYGEEIGMENINITADQVQDPFGKYNLSNSRDPQRSPMQWNSDMNTGFNNLTN 525

fugu_rubripes_Slc3a1 LPGTPNTYYGEELGMENINITG--SQDPAGKYNMRASRDPQRSPMQWSDDINAGFNNKTN 516

spotted_green_pufferfish_Slc3a1 LPGTPSTYYGEELGMENINITE--SQDPAGKHD-------ARAPMQWSGGLNAGFNNKTN 488

tropical_clawed_frog_Slc3a1 LPGTPTTYYGEELGMEDGSPNVDT--DKPEEYNP--VEYPEKTPMQWDSSENAGFSG-AN 531

chicken_SLC3A1 LPGTPITYYGEEIGMENIAS---E--NVT---------SPEKSPMQWNGNLNAGFTK-GN 516

mouse_Slc3a1 LPGTPITYYGEEIGMGDISV---T--NFNESYDS--TTLVSKSPMQWDNSSNAGFTE-AN 522

cow_SLC3A1 LPGTPITYYGEEIGMRNILA---A--NLNETYDA--GTLFSKSPMQWDNSSNAGFSE-GN 523

human_SLC3A1 LPGTPITYYGEEIGMGNIVA---A--NLNESYDI--NTLRSKSPMQWDNSSNAGFSE-AS 523

rhesus_SLC3A1 LPGTPITYYGEEIGMGNIVA---I--NLNESYDT--NTLRSKSPMQWDNSSNAGFSE-AS 523

***** ******:** : : ::****.. *:**. .

zebrafish_Slc3a1 GTWLDIASDYRTVNVEVQQDDTSSTISQYRALSLLRSSNVILSRGWFCYVWNDVNVFAYL 577

medaka_Slc3a1 ITWLPVHPDYKSVNVEVQKDSEGSTMFQYNFLNSLRQAELPFLRGWFCYVQADANIFSYL 585

fugu_rubripes_Slc3a1 VTWLPLHPDYSHVNVEVQKTDEGSVLAQYRFLSILRQSELPLYRGWFCYIYADADVFAYL 576

spotted_green_pufferfish_Slc3a1 VTWLPVHPAYKRVNVEVQKADEGSVLAQYRFLNILRQSELPLQRGWFCFVYADAHVFAYL 548

tropical_clawed_frog_Slc3a1 KTWLSINPDYEAVNVEAQKNEQYSTLNLYRELNNLRNNELPLHRGWLCYTWSDLNVFAYV 591

chicken_SLC3A1 SSWIPVNSDYESVNAEVQMTRFNSTLNLYRELTLLRNSELPIHRGWMCSIWNDSDVFVYV 576

mouse_Slc3a1 HTWLPTNSDYHTVNVDVQKTQPSSALRLYQDLSLLHATELVLSRGWFCLLRDDSHSVVYT 582

cow_SLC3A1 HTWLPTSSDYHTVNVDVQKTQPRSALKLYQELSLLHANELLLGRGWFCFLGNYNHSIMYT 583

human_SLC3A1 NTWLPTNSDYHTVNVDVQKTQPRSALKLYQDLSLLHANELLLNRGWFCHLRNDSHYVVYT 583

rhesus_SLC3A1 YTWLPTNSDYHTVNVDVQKTQSRSALKLYQDLSLLHANELLLNRGWFCHLRNDSHYVVYT 583

:*: * **.:.* *.: *. *. *: :: : ***:* . . *

zebrafish_Slc3a1 RELDGLSKGFLVVLNFGKE-TTTDL-SSVKELPDSLTLHLSTESITQT-EFPKSRIPTSQ 634

medaka_Slc3a1 RELDGHKEAYLMVLNFGKQSATTDL-SSVRELPDQLKVLMSTNSINNGKVFQKSGIQTEA 644

fugu_rubripes_Slc3a1 RELDGLDQAFLIVLNFGTKSKMTDL-SAVPELPQQLRVRMSTNRANDGKLLQKSHIQTEA 635

spotted_green_pufferfish_Slc3a1 RELDGLDRAFLIVLNFGPGWKTTDL-SAVAELPQQLRVRMSTVRANDGKLLQKSEIQTQA 607

tropical_clawed_frog_Slc3a1 RELDGLNKVFMMVLNFGGA-STINMRQEVPDLPAEAKIRLSTDGSRVGKAVNTGSIETKP 650

chicken_SLC3A1 RELDGLDSVFMMVLNFGQE-STIDLKAIVPDLPSKAVVRLSTDFSNNGKAVDTTIIKTDV 635

mouse_Slc3a1 RELDGIDNVFLVVLNFGESSTVLNLQGIISDLPPELRIRLSTNSASKGSAVDTRAISLEK 642

cow_SLC3A1 RELDGINRIFLMVLNFGES-SVLNLKEMISNIPTRVRIRLSTNSAYGGREVDTHAVTLAS 642

human_SLC3A1 RELDGIDRIFIVVLNFGES-TLLNLHNMISGLPAKMRIRLSTNSADKGSKVDTSGIFLDK 642

rhesus_SLC3A1 RELDGIDRIFIVVLNFGES-TLLNLQNMISGLPATVRIRLSTNSANKGSEVDTSGIFLDK 642

***** . :::***** :: : :* : :** . . :

zebrafish_Slc3a1 GQGLLLEYSSSQKFHLN--HQSQCYVSEKACYLPALDILYQ-C 674

medaka_Slc3a1 GEGLVIRYSTYTRFHPS--HATECYVSEKACYLDTVGILYK-C 684

fugu_rubripes_Slc3a1 GEGLVIEYSSHTRFNPN--HLDECFISEKACYLKVLGLLYT-C 675

spotted_green_pufferfish_Slc3a1 GEGLVLEYSSHTRFHPS--HQKDCFISEKACYLEVIGLLYT-C 647

tropical_clawed_frog_Slc3a1 GEGLILEYKTNKPVHVKEAFKDKCFISQKACYTSAFDLLYSSC 693

chicken_SLC3A1 GEGLVLEYKTGKPVHNMEAFRENCFVAEKACYSSAFNLLYMNC 678

mouse_Slc3a1 GEGLVLEHSTKAPLHQQAAFRDRCFVSSRACYSSALDILYSSC 685

cow_SLC3A1 GEGLILEYNTRNLLHRQTAFKERCFVSNRACYSRVLNILYSLC 685

human_SLC3A1 GEGLIFEHNTKNLLHRQTAFRDRCFVSNRACYSSVLNILYTSC 685

rhesus_SLC3A1 GEGLILEHNTKNLLHRQTAFRDRCFVSNRACYSSVLNILYTSC 685

*:**::.:.: .: . *:::.:*** ...:** *

**Fig. S1.** Amino acid sequence alignment of teleost fish (zebrafish, medaka, fugu rubripes, and spotted green pufferfish), amphibian (tropical clawed frog), bird (chicken) and mammalian (human, rhesus, cow, mouse) Slc3a1-type proteins. Multiple sequence alignment was generated using ClustalO(1.2.4) at <https://www.ebi.ac.uk/Tools/msa/clustalo/>, with default parameters. The putative transmembrane domain is named I, and is indicated by gray-boxed double-headed broken arrows. Within each sequence, the topological location of the predicted transmembrane domain is specifically underlined. The black arrow (↓) points to a cysteine residue (marked in green) involved in disulfide bridge formation with Slc7a9. Other conserved cysteine residues are marked in red. Putative N-glycosylation sites are marked in light blue. Putative protein kinase C phosphorylation sites and cyclic adenosine monophosphate and cyclic guanosine monophosphate-dependent protein kinase phosphorylation sites (sometimes superimposed) are also marked (in purple and brown, respectively).

**<----------I-----**

zebrafish_Slc7a9 -MEEDLKKT-KANNGSVSRAV-EA------KNPDQPKAAVLHQDVGLLSGICLIVGTMIG 51

medaka_Slc7a9 MGEGSIKKR-QPHNGTTGPT----------EKEKTEKATALQKDVGLLSGICLIVGTMIG 49

fugu_rubripes_Slc7a9 MDKESIKERKESQNGSTGQTT-DS----QTKDGLAEKATVLQKNVGLLSGICLIVGTIIG 55

spotted_green_pufferfish_Slc7a9 ------QAQKEPQNDSTDQTT-GS----QTKNKAPERAAALQKNVGLFSGTCLIVGTIIG 49

tropical_clawed_frog_Slc7a9 MDETKPRKRKGLQNGDADQKSIQSQESNTMNLKKQARTFNFFRFVGLISGISLIVGTIIG 60

chicken_SLC7A9 MGEGSLRKRKEQDNRKEDGQSIQSQELQSMNL---------KKQVGLISGICMIVGTIIG 51

mouse_Slc7a9 MEETS------LRRRREDEKSTHSTELKTTSL---------QKEVGLLSGICIIVGTIIG 45

cow_SLC7A9 MEETS------LRKRRGDEKSIQSSEPKTTSL---------QKELGLFSGTCIIVGTIIG 45

human_SLC7A9 MGDTG------LRKRREDEKSIQSQEPKTTSL---------QKELGLISGISIIVGTIIG 45

rhesus_SLC7A9 MGDTG------LRKRREDEKSIQSQEPKTTNL---------QKELGLISGICIIVGTIIG 45

. . . : :**:** .:****:**

**------> <-------------II-------------> <--------**

zebrafish_Slc7a9 SGIFISPKAVLEGTGAVGPCLCVWAACGVLATLGALCYAELGTMIIKSGGEYPYLMEGFG 111

medaka_Slc7a9 SGIFISPKSVLLYSGSVGPCLLIWTACGVLSTLGALCYAELGTMITKSGGEYPYLMEAFG 109

fugu_rubripes_Slc7a9 SGIFISPKAVLLYSGAVGPCLLIWASCGVLSILGALCYAELGTTIVKSGGDYSYYLEAFH 115

spotted_green_pufferfish_Slc7a9 SGIFISPKAVLLYSGAVGPCLLIWAACGVLSILGALCYAELGTTITKSGGDYSYYLEAFH 109

tropical_clawed_frog_Slc7a9 SGIFISPKSVLSNTGAIGPCLIIWAVCGVIATLGALCFAELGTMITKSGGEYPYLMEAFG 120

chicken_SLC7A9 SGIFVSPKSVLANVGAVGPCLTIWAACGVLATLGALCFAELGTTITKSGGEYPYLMEAFG 111

mouse_Slc7a9 SGIFISPKSVLANTESVGPCLIIWAACGILATLGALCFAELGTMITKSGGEYPYLMEAFG 105

cow_SLC7A9 SGIFISPKSVLRNMEAVGPCLIIWTMCGVLATLGALCFAELGTMITKSGGEYPYLMEAFG 105

human_SLC7A9 SGIFVSPKSVLSNTEAVGPCLIIWAACGVLATLGALCFAELGTMITKSGGEYPYLMEAYG 105

rhesus_SLC7A9 SGIFISPKSVLSNTEAVGPCLIIWAVCGVLAMLGALCFAELGTMITKSGGEYPYLMEAYG 105

****:***:** ::**** :*: **::: *****:***** * ****:* * :*.:

**--------III----------------> ↓ <----------IV------**

zebrafish_Slc7a9 PVLAYLYSWTTIIVLKPSSFAIIALSCAEYASTPFYPGCTPPQVVTKCLAAACILIITLV 171

medaka_Slc7a9 SIVAYLYSWTTVIVLKPSSFAIIALSFAEYTATPFYPGCTPPTVIIKCLAIASLLIIVAI 169

fugu_rubripes_Slc7a9 PIVAFLFSWTTVIALKPSSLAIITLSFAEYASSPFFPGCSPPTIVIKFLAATAILLIVTV 175

spotted_green_pufferfish_Slc7a9 PIVAFLFSWTMVIVLKPSSLAIITLSFAEYVSSPFYPGCSPPIIITKFLAATAILLIVTV 169

tropical_clawed_frog_Slc7a9 PIPAFLFSWSSLIVMKPSSFAIICLSFAEYVSAAFYPGCDPPVVVVKCLAAAVILTITLV 180

chicken_SLC7A9 PIPAFLFSWASLLVIKPSSFAIICLSFAEYASAPFYPGCDPPPVVIKCLAAAAIVVITVV 171

mouse_Slc7a9 PIPAYLFSWTSLIVMKPSSFAIICLSFSEYVCAAFYSGCKPPAVVVKLLAAAAILFITTV 165

cow_SLC7A9 PIPAYLFSWSSLFVIKPSSFAIICLSFSEYVCAPFYSGCSPPQVVVKSLAAAAILLISTV 165

human_SLC7A9 PIPAYLFSWASLIVIKPTSFAIICLSFSEYVCAPFYVGCKPPQIVVKCLAAAAILFISTV 165

rhesus_SLC7A9 PIPAYLFSWVSLMVMKPSSFAIICLSFSEYVCTPFYVGCKPPVIVVKCLAAAAILFITTV 165

: *:*:** ::.:**:*:*** ** :**..: *: ** ** :: * ** : :: * :

**-----> <-------------V-------------> <------------VI-**

zebrafish_Slc7a9 NCLSVKLAYRVQNFFTAAKLLIIIIIVVSGIVMLAQGNTQNLRDPFAGATTSFGAIGLAF 231

medaka_Slc7a9 NCISVKLASYVQNFFTAAKLVIILVIVVAGIVLLAQGKTQNLSNAFDGSSMSFGAIGLAF 229

fugu_rubripes_Slc7a9 NSLSVTLANYVQNFFTAAKLFIILVIAIAGIVLLAQGKTENLSNAFDGASTSFGAIGLAF 235

spotted_green_pufferfish_Slc7a9 NSLSVRLASYVQNFFTTAKLLIIFVIVIAGVVMLAQGKTQNLSNAFEGASTSFGAIGLAF 229

tropical_clawed_frog_Slc7a9 NALSVKLASYVQNFFTAAKMIIVIIIIVSGIVLLAQGKTQNFENSFDGATISVGGISLAL 240

chicken_SLC7A9 NSLSVKLGSYLQNLLTAAKMVVVAIIAVTGIVLLAQGKTQNFQDSFKDSKISVSSIGLAF 231

mouse_Slc7a9 NALSVRLGSYVQNVFTAAKMVIVAIIIISGLVFLAQGNVKNFQNSFEGTQTSVGAISLAF 225

cow_SLC7A9 NALSVRLGSYVQNMFTAAKMVIVVIIIVSGLVLLAQGNTRNFENSFEGASLSVGSISLAL 225

human_SLC7A9 NSLSVRLGSYVQNIFTAAKLVIVAIIIISGLVLLAQGNTKNFDNSFEGAQLSVGAISLAF 225

rhesus_SLC7A9 NSLSVRLGSYVQNIFTAAKLVIVAIIIISGLVLLAQGNTKNFDNSFEGAQLSVGAISLAF 225

*.:** *. :**.:*:**:.:: :* ::*:*:****:..*: : * .: *...*.**:

**---------> <------------VII----------->**

zebrafish_Slc7a9 YNGLWAYDGWNQLNFITEELKNPYKNLPLAIIIGIPLVTVCYIMVNIAYFSVMTSTELLQ 291

medaka_Slc7a9 YNGLWAYDGWNQLNFITEELRNPYRNLPLAIIIGIPLVTVCYVLVNIAYFTVLTPSELLV 289

fugu_rubripes_Slc7a9 YNGFWAYDGWNQLNFITEELKNPHRNLPLAILIGISLVTVCYVLVNVAYFTVMTPSELLL 295

spotted_green_pufferfish_Slc7a9 YNGFWAYDGWNQLNFITEELKNPHRNLPLAILIGISLVSVCYVLVNVAYFTVMTPSELLL 289

tropical_clawed_frog_Slc7a9 YNGLWAYDGWNQLNYITEELKNPYRNLPMAIIIGIPLVIVCYILINISYFTVLTPTELLQ 300

chicken_SLC7A9 YNGLWAYDGWNQLNYITEELKNPYRNLPLSIIIGIPLVTICYVLINISYFTVMTSTELLQ 291

mouse_Slc7a9 YNGLWAYDGWNQLNYITEELRNPYRNLPMAIVIGIPLVTVCYILMNIAYFTVMTPTELLQ 285

cow_SLC7A9 YNGLWAYDGWNQLNYITEELRNPFRNLPLAIIIGIPLVTGCYILMNVSYFTVMTATELLQ 285

human_SLC7A9 YNGLWAYDGWNQLNYITEELRNPYRNLPLAIIIGIPLVTACYILMNVSYFTVMTATELLQ 285

rhesus_SLC7A9 YNGLWAYDGWNQLNYITEELRNPYRNLPLAIIIGIPLVTVCYILINVSYFTVMTATELLQ 285

***:**********:*****:**.:***::*:*** ** **:::*::**:*:* :***

**<------------VIII-----------> <**

zebrafish_Slc7a9 SSAVAVTFGDRVLYPLSWIVPVFVVCSTFGAANGSCFTAGRLTYVAGREGHMVKIMSYIS 351

medaka_Slc7a9 SPAVAVTFGDRVLYPLSWIVPLFVAFSTFGAANGSCFTAGRLAFVSGREGHMVKILSYVS 349

fugu_rubripes_Slc7a9 SPAVAITFGDRVFYPLSWVVPLFVAFSTFGAANGSCFTAGRISYVSSREGHMVQILSFIS 355

spotted_green_pufferfish_Slc7a9 SPAVAVTFGDRVFYPLSWIVPLFVAFSTFGAANGSCFTAGRLSYVSSREGHMVQILSFIS 349

tropical_clawed_frog_Slc7a9 SQAVAVTFGDRVLYPAAWVVPLFVAFSTIGAANGTCFTSGRLAYVAGREGHMLKFLSYIS 360

chicken_SLC7A9 SQAVAVTFGDRVLYPASWIIPLFVAFSTIGSANGVCFTAGRLVYVAGREGHMLEVLSYIS 351

mouse_Slc7a9 SQAVAVTFGDRVLYPASWVVPLFVAFSTIGAANGTCFTAGRLIYVAGREGHMLKVLSYIS 345

cow_SLC7A9 SQAVAVTFGDRVLYPASWIVPLFVAFSTIGAANGSCFTAGRLVFVAGREGHMLKVLSYIS 345

human_SLC7A9 SQAVAVTFGDRVLYPASWIVPLFVAFSTIGAANGTCFTAGRLIYVAGREGHMLKVLSYIS 345

rhesus_SLC7A9 SQAVAVTFGDRVLYPASWVVPLFVAFSTIGAANGSCFTAGRLIYVAGREGHMLKVLSYIS 345

* ***:******:** :*::*:**. **:*:*** ***:**: :*:.*****::.:*::*

**--------------IX-----------<->-----------X---------->**

zebrafish_Slc7a9 VKRYTPSPALMFNGIVSIIYIMPTDINTLINYFSFATWLFYGLTCLALIVMRFTRKDLKR 411

medaka_Slc7a9 LRRYTPSPALIFNGILSLFYIIPADINSLINYFSFAQWAFYGLTALSLIVMRFTRKELHR 409

fugu_rubripes_Slc7a9 LKHCTPSPAIIFNGLLAICYIIPADIGILINYFSFAQWGFYGMSALALIVMRFTRKDLHR 415

spotted_green_pufferfish_Slc7a9 LKHCTPSPAIIFNGLLAICYIIPADIGTLINYFSFAQWGFYGMSALALIVLRFTRKDLHR 409

tropical_clawed_frog_Slc7a9 VKRLTPSPAIVFYGIIGMIYIIPADIDTLINYFSFAVWLFYGLTIAALVVMRFTRKELKR 420

chicken_SLC7A9 VKRLTPAPAIIFHGAIAIIYIIPGDINTLINYFSFAVWIFYGLSVFALIVMRFTRKEFKR 411

mouse_Slc7a9 VKRLTPAPALIFYGIIAIIYIIPGDINSLVNYFSFAAWLFYGMTILGLVVMRFTRKDLER 405

cow_SLC7A9 VRRLTPAPAIMFHGMIAIIYIIPGDINSLVNYFSFAAWLFYGLTITGLIVMRFTRKELKR 405

human_SLC7A9 VRRLTPAPAIIFYGIIATIYIIPGDINSLVNYFSFAAWLFYGLTILGLIVMRFTRKELER 405

rhesus_SLC7A9 VKRLTPAPAIIFHGIIATIYIIPGDINSLVNYFSFAAWLFYGLTILGLIVMRFTRKELER 405

::: **:**::* * :. **:* **. *:****** * ***:: .*:*:*****::.*

**<-----------XI---------> <---------XII---------->**

zebrafish_Slc7a9 PVKVPIVIPALVVVVSCYLVLAPIIDKPEWEYLYCTMFIVGGLLLYVPFIHYKFNWTRRL 471

medaka_Slc7a9 PVRVPIVIAALIVVVSCYLVLAPIIDQPELEYLYCTIFIFSGLILYYPFVYLKPRWARRI 469

fugu_rubripes_Slc7a9 PVKVPIVLAFLLGLLSCYLVLAPIIDKPTIEYLYCSIFIFSGVILYYFFIHRKVKWAQRV 475

spotted_green_pufferfish_Slc7a9 PVKVPIVLAFLLALVSCYLVLAPIIDKPTIEYLYCSIFIFSGVILYYFFIYRKVKWAQRI 469

tropical_clawed_frog_Slc7a9 PIKVPIVIPIVMVLISIYLVLAPIIDKPELAYLYCVLFILSGLIVYFPFVHYKVKWAQKI 480

chicken_SLC7A9 PIRIPIIIPVIVTLISILLVLAPIITAPELAYLYCVLFILSGLIFYVLFVHFKFKWSQKI 471

mouse_Slc7a9 PIKVPLFIPIIVILVSLFLILAPIISEPAWEYLYCVLFILSGLIFYFLFVYYKFGWAQRI 465

cow_SLC7A9 PIKVPIFIPILVTLLSVFLVLAPIISAPAWEYLYCVLFMLSGLVFYFLFVYYKFAWAQKI 465

human_SLC7A9 PIKVPVVIPVLMTLISVFLVLAPIISKPTWEYLYCVLFILSGLLFYFLFVHYKFGWAQKI 465

rhesus_SLC7A9 PIKVPIVIPVLVTFISVFLILAPIISKPAWEYLYCVLFILSGLIFYFLFVHYKFGWAQKI 465

*:::*:.: :: .:* *:***** * **** :*:..*::.* *:: * *::::

zebrafish_Slc7a9 MRPFTMHLQLLLQVVPPEKIE---------- 492

medaka_Slc7a9 MRPITMHLQLFMEVVPPEDYEPEDPMKSVKF 500

fugu_rubripes_Slc7a9 SRPITTYLQLLMEVVPPEKTETD-------- 498

spotted_green_pufferfish_Slc7a9 SRVITTYLQLLMEVVPPEETKT--------- 491

tropical_clawed_frog_Slc7a9 TRPITMFIQMLMEVVPPEETKE--------- 502

chicken_SLC7A9 SEPITMHLQMLLEVVPPEEVAE--------- 493

mouse_Slc7a9 SRPVTKHLQMLMEVVPPEKDPE--------- 487

cow_SLC7A9 SKPLTMHLQMLMEVVPPEEAPE--------- 487

human_SLC7A9 SKPITMHLQMLMEVVPPEEDPE--------- 487

rhesus_SLC7A9 SKPITMHLQMLMEVVPPEEDPE--------- 487

. .* .:*::::*****.

**Fig. S2.** Amino acid sequence alignment of teleost fish (zebrafish, medaka, fugu rubripes, and spotted green pufferfish), amphibian (tropical clawed frog), bird (chicken), and mammalian (human, rhesus, cow, mouse) Slc7a9-type proteins. Multiple sequence alignment was generated using ClustalO(1.2.4) at <https://www.ebi.ac.uk/Tools/msa/clustalo/>, with default parameters. The putative transmembrane domains are named I to XII, and are indicated by gray-boxed double-headed broken arrows (with darker gray designating the core stretch of amino acids within each putative predicted transmembrane segment that is shared by at least two of the aligned sequences). Within each sequence, the topological location of each predicted transmembrane domain is specifically underlined. The black arrow (↓) points to a cysteine residue (marked in green) involved in disulfide bridge formation with Slc3a1. Other conserved cysteine residues are marked in red. Putative N-glycosylation sites are marked in light blue. The C-terminal VPP sequence shown to be responsible for the endoplasmic reticulum-to-Golgi trafficking of a heterodimer (Sakamoto et al. 2009) is marked in blue. Putative protein kinase C phosphorylation sites and cyclic adenosine monophosphate and cyclic guanosine monophosphate-dependent protein kinase phosphorylation sites (sometimes superimposed) are also marked (in purple and brown, respectively).


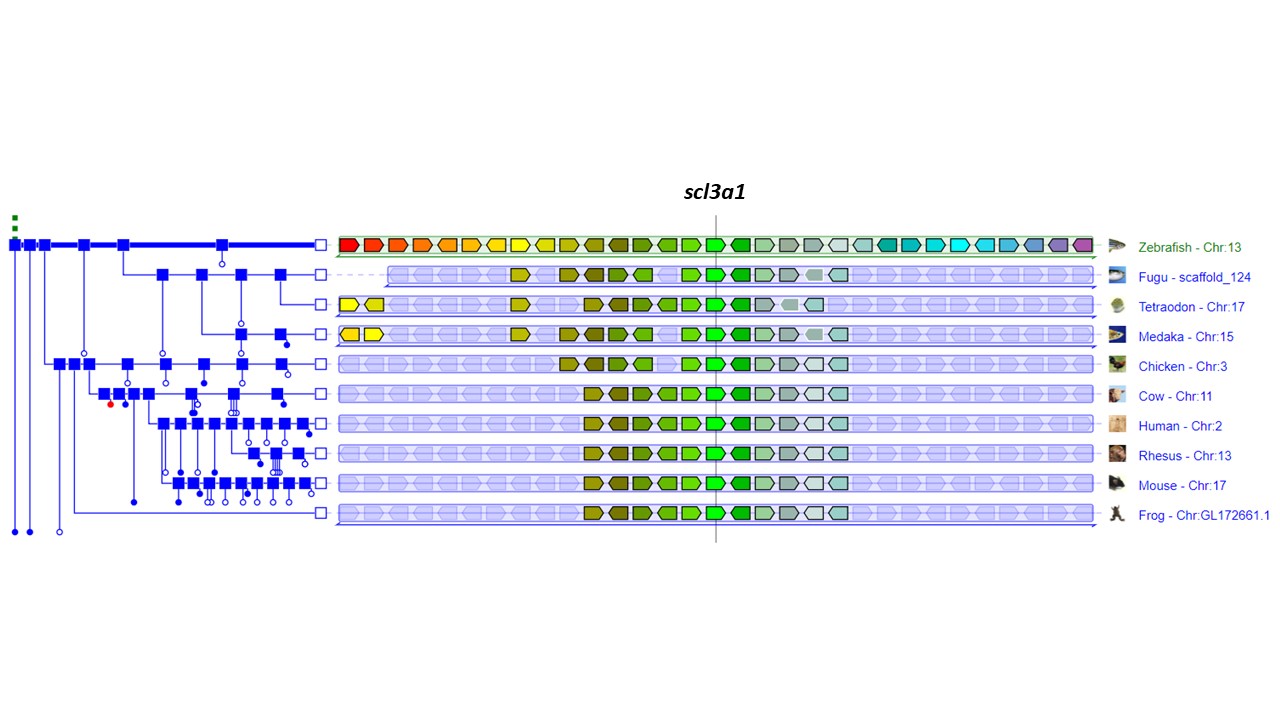


**Fig. S3. Representation of the *slc3a1* reference gene in the zebrafish genome.** The left part is the phylogenetic tree of this reference gene. On the right, the reference gene and its homologous copies in other species (*i.e.*, human, rhesus, mouse, cow, chicken, tropical clawed frog, medaka, fugu rubripes, and spotted green pufferfish) are in the center surrounded by their neighboring genes in their respective genomes. Genes of the same color are homologs. Results were obtained by consulting Genomicus (database version: 93.01; Nguyen et al. 2018). Genomicus analysis for zebrafish *slc3a1* shows that it is in conserved synteny with the other species; *e.g.*, with the following human genes on chromosome 2 (corresponding zebrafish genes on chromosome 13 are mentioned in brackets): *DYNC2LI1 (dync2li1)* | *ABCG5 (abcg5)* | *ABCG8 (abcg8)* | *LRPPRC (*[*lrpprc*](im:7150924)*)* | *PPM1B (ppm1ba)* | *SLC3A1 (slc3a1)* | *PREPL (prepl)* | *CAMKMT (si:ch73-54n14.2) (camkmt)* | *SIX3 (six3a)* | *SIX2 (six2a)* | *SRBD1 (si:ch211-117n7.8) (srbd1).*


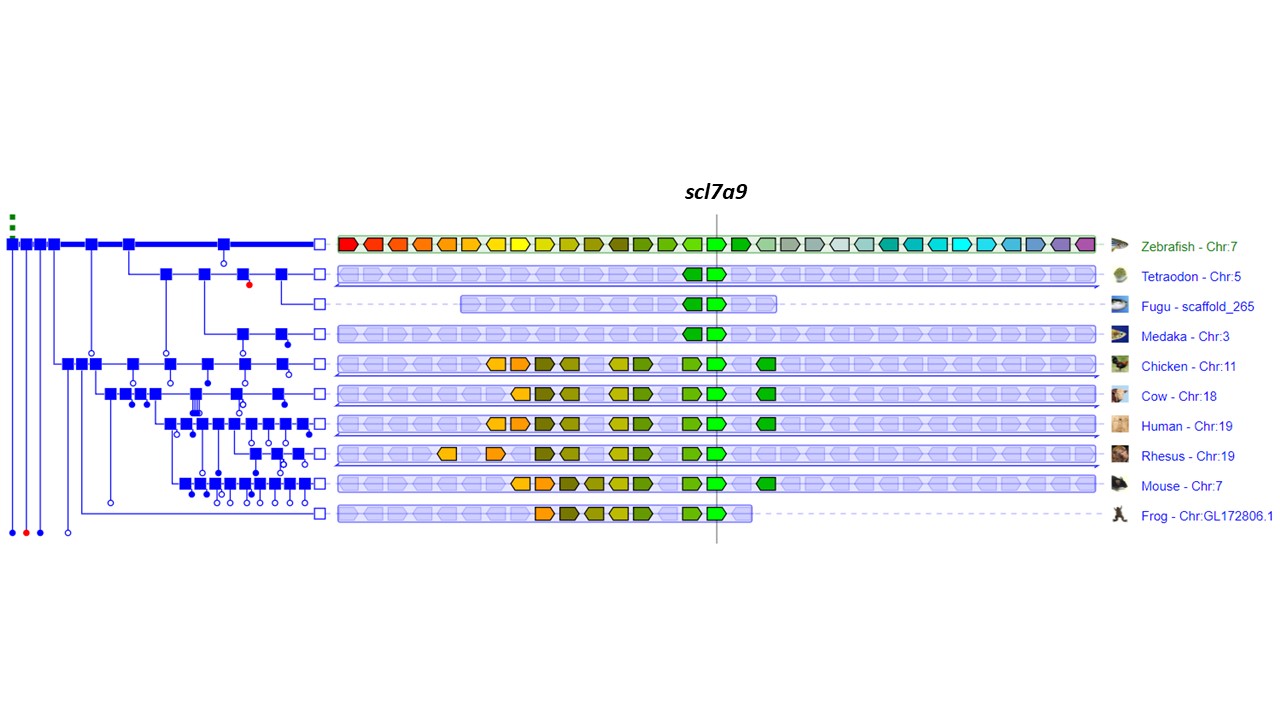


**Fig. S4. Representation of the *slc7a9* reference gene in the zebrafish genome.** The left part is the phylogenetic tree of this reference gene. On the right, the reference gene and its homologous copies in other species (*i.e.*, human, rhesus, mouse, cow, chicken, tropical clawed frog, medaka, fugu rubripes, and spotted green pufferfish) are in the center surrounded by their neighboring genes in their respective genomes. Genes of the same color are homologs. Results were obtained by consulting Genomicus (database version: 93.01; Nguyen et al. 2018). Genomicus analysis for zebrafish *slc7a9* shows that it is in conserved synteny with the other species; *e.g.*, with the following human genes on chromosome 19 (corresponding zebrafish genes on chromosome 7 are mentioned in brackets): *CHST8 (-)* | *PEPD (-) | - (cebpa)* | *- (cebpg)* | *CEBPG (-) | CEBPA (-)* | *- (abcc12)* | *- (si:dkey-78a14.4)* | *- (cnep1r1)* | *SLC7A10 (-)* | *LPR3 (-)* | *WDR88 (-)* | *GPATCH1 (gpatch1) | - (lrp3)* | *- (slc7a10a)* | *RHPN2 (rhpn2)* | *FAAP24 (-)* | *CEP89 (*[*cep89*](im:7150924)*)* | *- (zgc:165481)* | *SLC7A9 (slc7a9)* | *TDRD12 (-)* | *NUTD19 (nutd19).*

**Appendix I**

**Slc3a1 Sequences**

>human_SLC3A1|ENSP00000260649|Q07837 (SLC31_HUMAN)

MAEDKSKRDSIEMSMKGCQTNNGFVHNEDILEQTPDPGSSTDNLKHSTRGILGSQEPDFKGVQPYAGMPK

EVLFQFSGQARYRIPREILFWLTVASVLVLIAATIAIIALSPKCLDWWQEGPMYQIYPRSFKDSNKDGNG

DLKGIQDKLDYITALNIKTVWITSFYKSSLKDFRYGVEDFREVDPIFGTMEDFENLVAAIHDKGLKLIID

FIPNHTSDKHIWFQLSRTRTGKYTDYYIWHDCTHENGKTIPPNNWLSVYGNSSWHFDEVRNQCYFHQFMK

EQPDLNFRNPDVQEEIKEILRFWLTKGVDGFSLDAVKFLLEAKHLRDEIQVNKTQIPDTVTQYSELYHDF

TTTQVGMHDIVRSFRQTMDQYSTEPGRYRFMGTEAYAESIDRTVMYYGLPFIQEADFPFNNYLSMLDTVS

GNSVYEVITSWMENMPEGKWPNWMIGGPDSSRLTSRLGNQYVNVMNMLLFTLPGTPITYYGEEIGMGNIV

AANLNESYDINTLRSKSPMQWDNSSNAGFSEASNTWLPTNSDYHTVNVDVQKTQPRSALKLYQDLSLLHA

NELLLNRGWFCHLRNDSHYVVYTRELDGIDRIFIVVLNFGESTLLNLHNMISGLPAKMRIRLSTNSADKG

SKVDTSGIFLDKGEGLIFEHNTKNLLHRQTAFRDRCFVSNRACYSSVLNILYTSC

>rhesus_SLC3A1|ENSMMUP00000008308|F7HIT7 (F7HIT7_MACMU)

MAEDKSKRDSIGMNMKGCQTNNGFVHNEDILEQTPDTGSSADNLQHSTTSILGSQEPDFKGVQPYAGMPK

EVLFQFSGQARYRIPREILFWLAVASVLVLIAATVAIIVLSPKCLDWWQEGPMYQIYPRSFKDSNKDGNG

DLKGIQDKLDYITALNVKTVWITSFYKSSLKDFRYGVEDFRQVDSIFGTMEDFENLVAAIHDKGLKLIID

FIPNHTSDKHTWFQLSRTRTGKYTDYYIWHDCTHENGTTVPPNNWLSVYGNSSWHFDEVRNQCYFHQFMK

EQPDLNFRNPDVQEEIKEILQFWLTKGVDGFSFDAVKFLLEAKHLRDEIQVNKTQIPNTVTQYSELYHDF

TTTQVGMHDIVRSFRQTMDQYSTEPGRYRFMGTEAYAETIDRTVMYYGLPFIQEADFPFNNYLTMLDTVS

GNSVYEVITSWMENMPEGKWPNWMIGGPDSSRLTSRLGNEYVNVMNMLLFTLPGTPITYYGEEIGMGNIV

AINLNESYDTNTLRSKSPMQWDNSSNAGFSEASYTWLPTNSDYHTVNVDVQKTQSRSALKLYQDLSLLHA

NELLLNRGWFCHLRNDSHYVVYTRELDGIDRIFIVVLNFGESTLLNLQNMISGLPATVRIRLSTNSANKG

SEVDTSGIFLDKGEGLILEHNTKNLLHRQTAFRDRCFVSNRACYSSVLNILYTSC

>mouse_Slc3a1|ENSMUSP00000024944|Q91WV7 (SLC31_MOUSE)

MDEDKGKRDPIQMSLKGCRTNNGFVQNEDIPEQDPDPGSRDTPQPNAVSIPAPEEPHLKAVRPYAGMPKE

VLFQFSGQARYRVPREILFWLTVVSVFLLIGATIAIIVISPKCLDWWQAGPIYQIYPRSFKDSDKDGNGD

LKGIQEKLDYITALNIKTLWITSFYKSSLKDFRYAVEDFKEIDPIFGTMKDFENLVAAIHDKGLKLIIDF

IPNHTSDKHPWFQSSRTRSGKYTDYYIWHNCTHVNGVTTPPNNWLSVYGNSSWHFDEVRKQCYFHQFLKE

QPDLNFRNPAVQEEIKEIITFWLSKGVDGFSFDAVKFLLEAKDLRNEIQVNTSQIPDTVTHYSELYHDFT

TTQVGMHDIVRDFRQTMNQYSREPGRYRFMGAEASAESIERTMMYYGLPFIQEADFPFNKYFTTIGTLSG

HTVYEVITSWMENMPEGKWPNWMTGGPETPRLTSRVGSEYVNAMHMLLFTLPGTPITYYGEEIGMGDISV

TNFNESYDSTTLVSKSPMQWDNSSNAGFTEANHTWLPTNSDYHTVNVDVQKTQPSSALRLYQDLSLLHAT

ELVLSRGWFCLLRDDSHSVVYTRELDGIDNVFLVVLNFGESSTVLNLQGIISDLPPELRIRLSTNSASKG

SAVDTRAISLEKGEGLVLEHSTKAPLHQQAAFRDRCFVSSRACYSSALDILYSSC

>cow_SLC3A1|ENSBTAP00000023161|Q3SZF7 (Q3SZF7_BOVIN)

MAEDKSKRDSIGLNAKEGQTNNGFVQNEDIRETDLDPSSPVVGPQHNTVDILGPGEPDVKDVRPYAGMPK

EVLFQFSGQACYRIPREVLFWLTVASVLVLIAATIAIIAISPKCLDWWQAGPMYQIYPRSFRDSNKDGDG

DLKGIQDKLDYITTLNIKTVWITSFYKSSLKDFRHGVEDFREIDPIFGTMKDFENLVAAIHDKGLKLIID

FIPNHTSDKHAWFQWSRNQTGKYTDYYIWHDCNRENGTTIPPNNWLSVYGNSSWHFDEVRKQCYFHQFMK

EQPDLNFRNPDVQEEIKEIIQFWLSKGVDGFSFDALPFLLEAKHLRDEAQVNKTQIPDMVTHYSQLHHDF

TTTQVGMHDIVRSFRQTMNQYSREPGRYRFMGTEAHGESITKTMVYYGLPFIQEADFPFNSYLSKLDKPS

GNSVSEIITSWMENMPEGKWPNWMTGGPDSVRLTSRLGEKYVNIMNMLVFTLPGTPITYYGEEIGMRNIL

AANLNETYDAGTLFSKSPMQWDNSSNAGFSEGNHTWLPTSSDYHTVNVDVQKTQPRSALKLYQELSLLHA

NELLLGRGWFCFLGNYNHSIMYTRELDGINRIFLMVLNFGESSVLNLKEMISNIPTRVRIRLSTNSAYGG

REVDTHAVTLASGEGLILEYNTRNLLHRQTAFKERCFVSNRACYSRVLNILYSLC

>chicken_SLC3A1|ENSGALP00000016193|E1C011 (E1C011_CHICK)|see_also_XP_004935427.1

MVDKELKGLPMELSEKGGVENNGFVQNEAFDDRDTDTSSQDEIPKTCAVDVSPAAVAEPALQPYAGMPKE

VLLKFSSQARYRVTREILFWLTIAAAVLLVSATIAIIALSPKCLDWWQDGPIYQVYPRSFKDSNMDGNGD

LKGIQEKLDHITHLNIKTIWITSFYKSPLKDAGYGAEDFYDIDPMFGSMRDFEDLLASIHDRGLKVIMDF

IPNHTSDKHQWFQLSRNRTGKYTDYYIWQDCVQAGAAISAPNNWVSVYGNSSWQYDDVRKQCYFHQFGKE

QPDLNFRNPAVQQEIHDVIKFWLGKGVDGFNFIAVKFLLEATHLRDEPQVNKSQNPESITAYSELYHDYT

TTQVGMHDIIRSFRQTMDQYSSEPGRYRFMGSDSDEKEDIEATMMYYGTTFVQEADFPFNLHLINMKNLS

GNSVFEAVNMWMKNMPEGKWPNWAVGSPNTARISSRFGSEYVRVINMLLLTLPGTPITYYGEEIGMENIA

SENVTSPEKSPMQWNGNLNAGFTKGNSSWIPVNSDYESVNAEVQMTRFNSTLNLYRELTLLRNSELPIHR

GWMCSIWNDSDVFVYVRELDGLDSVFMMVLNFGQESTIDLKAIVPDLPSKAVVRLSTDFSNNGKAVDTTI

IKTDVGEGLVLEYKTGKPVHNMEAFRENCFVAEKACYSSAFNLLYMNC

>tropical_clawed_frog_Slc3a1|ENSXETP00000005991|Q0V9A1 (Q0V9A1_XENTR)

MEEDPNSVELKQKAGHENNGFISDHDGKEEGKPEKVEIRRNSKEEDTHIYSVRIDEIPENAPPLKPYAGM

PKDVLLQFSNQPCYRIAREIIFWLIIVATLAIIAATIAIIAISPKCLDWWQRSPIYQVYPKSFKDSNNDG

SGDLKGVQEKIDHFIYLDVKNIWVAPFYKSSLKDFNYAVDDHMEVDPTFGTMADFDSMISAMHDKGLKLI

IDLIPNHTSNKHKWFQLSRNRTDKYTDYYIWHDCAQVGGVRVPPNNWVSVYGDSAWEYDVTRNQCYLHQF

RKEQPDLNFNNPDVHEEILNIIKFWLGKGVDGFTINSAKFILEAEHFRDEPQVNKLQDPATISNYAELFH

DYTTTQVGMHDIIRNFRQTINKYSREPGRYRFMGTESNDQPAIDKTMLYYGNSFIQEADFPLNSYLLDLS

RTNLNGLSIFSNVDLWMKSMPSGKWPSWMVGGPSYSRISSRVGRQYINVMNMLLLTLPGTPTTYYGEELG

MEDGSPNVDTDKPEEYNPVEYPEKTPMQWDSSENAGFSGANKTWLSINPDYEAVNVEAQKNEQYSTLNLY

RELNNLRNNELPLHRGWLCYTWSDLNVFAYVRELDGLNKVFMMVLNFGGASTINMRQEVPDLPAEAKIRL

STDGSRVGKAVNTGSIETKPGEGLILEYKTNKPVHVKEAFKDKCFISQKACYTSAFDLLYSSC

>zebrafish_Slc3a1|ENSDARP00000114538|F1QEA9 (F1QEA9_DANRE)

MSLTRVTNIDAVELQEGIQNAAFREDDDDNNGDIVCIRRVSSLPEESEYTQIKPYAGMPKEVLMLYSNKP

CYRIPREVIFWLVIACTLALIAMTAAIVALSPRCMSWWQLSPIYQVYPRSFKDSNADGVGDLRGIKEKLS

HFEYLNIKAIWISPFYKSPMRDFGYDVEDFRDVDPLFGTMEDFDDLLTSMHDKGLKLIMDYIPNHTSDKH

VWFQLSRNYTEPYTDYYIWVNCTADKHPNNWVSVFGNSTWEYDEIRQQCYFHQFLKEQPDLNYRNPLVLQ

EMTDIIHFWLKKGVDGFRMDAVKHMLEATHLRDEPQVNPDQDPSTVDTEFELYHDYTYTQAGLHEILTDW

RIQMDTYSREPGRYRFMVMESYDYEEIDKTMRYYGTNYAKESDFPFNFYLLDLPDNLSGNYAKSIVERWM

SNMPKGKWPNWVVGNHDKPRIGSSAGKEYVRALNMLLLTLPGTPTTYYGEEIGMVDVNISVIQDPAGQYD

PSKSRDPQRTPMQWNNELNAGFSESLNGTWLDIASDYRTVNVEVQQDDTSSTISQYRALSLLRSSNVILS

RGWFCYVWNDVNVFAYLRELDGLSKGFLVVLNFGKETTTDLSSVKELPDSLTLHLSTESITQTEFPKSRI

PTSQGQGLLLEYSSSQKFHLNHQSQCYVSEKACYLPALDILYQC

>medaka_Slc3a1|ENSORLP00000005633|H2LI15 (H2LI15_ORYLA)

MSYIRKDSGSVELADGIRNLGFQDADVVTDFPPHSSPRDAVERKDSAAEIDDSVYAQVEPYAGMPKEVLL

LYSSQARYRVPREVLFWLTVCCTLALVAVTVAIIALSPPCLGWWQISPVYQVYPRSFKDSDGDGVGDLRG

IKEKLDHFHSLNIKSIWISPFYRSPMKDFGYDVEDFRDVDPLFGTMEDFEELLAEMHKNGLKLIMDFIPN

HSSDRHRWFNLSRTRDPLYEDYYVWADCNATKKPNNWVSIFGNSSWTYDEVRGQCYLHQFLKEQPDLNMR

NPAVRKEIIDIIHFWLSKGVDGFRMDAVKHMLEATHLRDEPQVDPNKPPEEVTTEWDLFHDYTTSQVGLH

DILREFRAEMEPYSREPGKYRFMVTESYDYEEVEKTMMYYGTPLVKESDFPFNFYLLDLPQNASGLWAKH

LVDLWMSNMPRGKWPNWVVGNHDRSRIASSAGKMYVRVINMLLLTLPGTPTTYYGEEIGMENINITADQV

QDPFGKYNLSNSRDPQRSPMQWNSDMNTGFNNLTNITWLPVHPDYKSVNVEVQKDSEGSTMFQYNFLNSL

RQAELPFLRGWFCYVQADANIFSYLRELDGHKEAYLMVLNFGKQSATTDLSSVRELPDQLKVLMSTNSIN

NGKVFQKSGIQTEAGEGLVIRYSTYTRFHPSHATECYVSEKACYLDTVGILYKC

>fugu_rubripes_Slc3a1|ENSTRUP00000019541|H2T4F3 (H2T4F3_TAKRU)

IWNAVMALVRYHTNPGFEDVEVGETTAELNTVPEEKGCSAAGTRDVDASEEYTQIKPYPGMPKEVLLLYS

SQARYRVPREIIFWLTVACTAALVGLTATVIALSPRCLSWWQTSPMYQLYPRSFKDSDGDGIGDLKGILQ

KLDHFQYLNIKSVWIGPLYRSPMKDFGYDVEDFRSISPEFGTMQDFEELLAEMHNRGLNLIMDFIPNHTS

DRHPWFILSRTRDPQYEDYYIWADCNQTAPRPNNWVSVFGNTSWAYDEVRGQCYLHQFFKEEPDLNLRNP

RVRKEMIDIIRFWLEKGVDGFRIGSAKYALEAAHLRDEPQVDPNKPSVTSDEDLHRDYTTSQLGLHDLLR

EWRAEMDAYSHEPGRYRLMVTESYDYHEVEKTMMYYSTPLAKESDLPFNFYLLDLPQNNSGLWVKQLVDL

WMANMPRGQWANWVVGNHDKPRIASTAGQTYVALINMLLLTLPGTPNTYYGEELGMENINITGSQDPAVQ

RASRDPQRSPMQWSDDINAGFNNKTNVTWLPLHPDYSHVNVEVQKTDEGSVLAQYRFLSILRQSELPLYR

GWFCYIYADADVFAYLRELDGLDQAFLIVLNFGTKSKMTDLSAVPELPQQLRVRMSTNRANDGKLLQKSH

IQTEAGEGLVIEYSSHTRFNPNHLDECFISEKACYLKVLGLLYTC

>spotted_green_pufferfish_Slc3a1|ENSTNIP00000010276|H3CPU3 (H3CPU3_TETNG)

ELPTDPRERASVAGPRAEEEDPSAEYAQIKPYAGMPREVLLLYSSQARYRVPREIIFWLTVACTAALVGL

TVTVIVLSPRCLSWWQTSPVYQLYPRSFKDSDGDGVGDLKGILEKLDHFQYLNIKSVWIGPLYRSPMRDF

GYDVEDFRSISPEFGTMQDFEELLAEMHNRGLNLIMDFIPNHTSDRHPWFNLSRTGDPHYRDYYVWADCN

QTAARPNNWVSVFGNSSWTYDDVRGQCYLHQFFKEQPDLNLRNPSVRREMIDIIRFWLEKGVDGFRIGSV

KYVLEAAHLRDEPQVDPKKPAESVTADKDLHRDYTTSQLGLHDLLREWRAEMDAYSREPGRYRLMVTESY

DDHEVEKTMMYYSSPLVKESDFPFNFYLLDLPQNSSGLWARQLVHLWMASMPRGQWANWLVGNHDKPRIA

SAAGQTYVALFNMLLLTLPGTPSTYYGEELGMENINITESQDPAGKHDARAPMQWSGGLNAGFNNKTNVT

WLPVHPAYKRVNVEVQKADEGSVLAQYRFLNILRQSELPLQRGWFCFVYADAHVFAYLRELDGLDRAFLI

VLNFGPGWKTTDLSAVAELPQQLRVRMSTVRANDGKLLQKSEIQTQAGEGLVLEYSSHTRFHPSHQKDCF

ISEKACYLEVIGLLYTC

**Slc7a9 sequences**

>human_SLC7A9|ENSP00000023064|P82251 (BAT1_HUMAN)

MGDTGLRKRREDEKSIQSQEPKTTSLQKELGLISGISIIVGTIIGSGIFVSPKSVLSNTEAVGPCLIIWA

ACGVLATLGALCFAELGTMITKSGGEYPYLMEAYGPIPAYLFSWASLIVIKPTSFAIICLSFSEYVCAPF

YVGCKPPQIVVKCLAAAAILFISTVNSLSVRLGSYVQNIFTAAKLVIVAIIIISGLVLLAQGNTKNFDNS

FEGAQLSVGAISLAFYNGLWAYDGWNQLNYITEELRNPYRNLPLAIIIGIPLVTACYILMNVSYFTVMTA

TELLQSQAVAVTFGDRVLYPASWIVPLFVAFSTIGAANGTCFTAGRLIYVAGREGHMLKVLSYISVRRLT

PAPAIIFYGIIATIYIIPGDINSLVNYFSFAAWLFYGLTILGLIVMRFTRKELERPIKVPVVIPVLMTLI

SVFLVLAPIISKPTWEYLYCVLFILSGLLFYFLFVHYKFGWAQKISKPITMHLQMLMEVVPPEEDPE

>rhesus_SLC7A9|ENSMMUP00000031688|F6XDS9 (F6XDS9_MACMU)

MGDTGLRKRREDEKSIQSQEPKTTNLQKELGLISGICIIVGTIIGSGIFISPKSVLSNTEAVGPCLIIWA

VCGVLAMLGALCFAELGTMITKSGGEYPYLMEAYGPIPAYLFSWVSLMVMKPSSFAIICLSFSEYVCTPF

YVGCKPPVIVVKCLAAAAILFITTVNSLSVRLGSYVQNIFTAAKLVIVAIIIISGLVLLAQGNTKNFDNS

FEGAQLSVGAISLAFYNGLWAYDGWNQLNYITEELRNPYRNLPLAIIIGIPLVTVCYILINVSYFTVMTA

TELLQSQAVAVTFGDRVLYPASWVVPLFVAFSTIGAANGSCFTAGRLIYVAGREGHMLKVLSYISVKRLT

PAPAIIFHGIIATIYIIPGDINSLVNYFSFAAWLFYGLTILGLIVMRFTRKELERPIKVPIVIPVLVTFI

SVFLILAPIISKPAWEYLYCVLFILSGLIFYFLFVHYKFGWAQKISKPITMHLQMLMEVVPPEEDPE

>mouse_Slc7a9|ENSMUSP00000032703|Q9QXA6 (BAT1_MOUSE)

MEETSLRRRREDEKSTHSTELKTTSLQKEVGLLSGICIIVGTIIGSGIFISPKSVLANTESVGPCLIIWA

ACGILATLGALCFAELGTMITKSGGEYPYLMEAFGPIPAYLFSWTSLIVMKPSSFAIICLSFSEYVCAAF

YSGCKPPAVVVKLLAAAAILFITTVNALSVRLGSYVQNVFTAAKMVIVAIIIISGLVFLAQGNVKNFQNS

FEGTQTSVGAISLAFYNGLWAYDGWNQLNYITEELRNPYRNLPMAIVIGIPLVTVCYILMNIAYFTVMTP

TELLQSQAVAVTFGDRVLYPASWVVPLFVAFSTIGAANGTCFTAGRLIYVAGREGHMLKVLSYISVKRLT

PAPALIFYGIIAIIYIIPGDINSLVNYFSFAAWLFYGMTILGLVVMRFTRKDLERPIKVPLFIPIIVILV

SLFLILAPIISEPAWEYLYCVLFILSGLIFYFLFVYYKFGWAQRISRPVTKHLQMLMEVVPPEKDPE

>cow_SLC7A9|ENSBTAP00000034433|Q3ZCL6 (Q3ZCL6_BOVIN)

MEETSLRKRRGDEKSIQSSEPKTTSLQKELGLFSGTCIIVGTIIGSGIFISPKSVLRNMEAVGPCLIIWT

MCGVLATLGALCFAELGTMITKSGGEYPYLMEAFGPIPAYLFSWSSLFVIKPSSFAIICLSFSEYVCAPF

YSGCSPPQVVVKSLAAAAILLISTVNALSVRLGSYVQNMFTAAKMVIVVIIIVSGLVLLAQGNTRNFENS

FEGASLSVGSISLALYNGLWAYDGWNQLNYITEELRNPFRNLPLAIIIGIPLVTGCYILMNVSYFTVMTA

TELLQSQAVAVTFGDRVLYPASWIVPLFVAFSTIGAANGSCFTAGRLVFVAGREGHMLKVLSYISVRRLT

PAPAIMFHGMIAIIYIIPGDINSLVNYFSFAAWLFYGLTITGLIVMRFTRKELKRPIKVPIFIPILVTLL

SVFLVLAPIISAPAWEYLYCVLFMLSGLVFYFLFVYYKFAWAQKISKPLTMHLQMLMEVVPPEEAPE

>chicken_SLC7A9|ENSGALP00000007521|E1BT87 (E1BT87_CHICK)|see_also_NP_001186062.1

MGEGSLRKRKEQDNRKEDGQSIQSQELQSMNLKKQVGLISGICMIVGTIIGSGIFVSPKSVLANVGAVGP

CLTIWAACGVLATLGALCFAELGTTITKSGGEYPYLMEAFGPIPAFLFSWASLLVIKPSSFAIICLSFAE

YASAPFYPGCDPPPVVIKCLAAAAIVVITVVNSLSVKLGSYLQNLLTAAKMVVVAIIAVTGIVLLAQGKT

QNFQDSFKDSKISVSSIGLAFYNGLWAYDGWNQLNYITEELKNPYRNLPLSIIIGIPLVTICYVLINISY

FTVMTSTELLQSQAVAVTFGDRVLYPASWIIPLFVAFSTIGSANGVCFTAGRLVYVAGREGHMLEVLSYI

SVKRLTPAPAIIFHGAIAIIYIIPGDINTLINYFSFAVWIFYGLSVFALIVMRFTRKEFKRPIRIPIIIP

VIVTLISILLVLAPIITAPELAYLYCVLFILSGLIFYVLFVHFKFKWSQKISEPITMHLQMLLEVVPPEE

VAE

>tropical_clawed_frog_Slc7a9|ENSXETP00000039600|F7C436 (F7C436_XENTR)

MDETKPRKRKGLQNGDADQKSIQSQESNTMNLKKQARTFNFFRFVGLISGISLIVGTIIGSGIFISPKSV

LSNTGAIGPCLIIWAVCGVIATLGALCFAELGTMITKSGGEYPYLMEAFGPIPAFLFSWSSLIVMKPSSF

AIICLSFAEYVSAAFYPGCDPPVVVVKCLAAAVILTITLVNALSVKLASYVQNFFTAAKMIIVIIIIVSG

IVLLAQGKTQNFENSFDGATISVGGISLALYNGLWAYDGWNQLNYITEELKNPYRNLPMAIIIGIPLVIV

CYILINISYFTVLTPTELLQSQAVAVTFGDRVLYPAAWVVPLFVAFSTIGAANGTCFTSGRLAYVAGREG

HMLKFLSYISVKRLTPSPAIVFYGIIGMIYIIPADIDTLINYFSFAVWLFYGLTIAALVVMRFTRKELKR

PIKVPIVIPIVMVLISIYLVLAPIIDKPELAYLYCVLFILSGLIVYFPFVHYKVKWAQKITRPITMFIQM

LMEVVPPEETKE

>zebrafish_Slc7a9|ENSDARP00000091252|F1QGJ2 (F1QGJ2_DANRE)

MEEDLKKTKANNGSVSRAVEAKNPDQPKAAVLHQDVGLLSGICLIVGTMIGSGIFISPKAVLEGTGAVGP

CLCVWAACGVLATLGALCYAELGTMIIKSGGEYPYLMEGFGPVLAYLYSWTTIIVLKPSSFAIIALSCAE

YASTPFYPGCTPPQVVTKCLAAACILIITLVNCLSVKLAYRVQNFFTAAKLLIIIIIVVSGIVMLAQGNT

QNLRDPFAGATTSFGAIGLAFYNGLWAYDGWNQLNFITEELKNPYKNLPLAIIIGIPLVTVCYIMVNIAY

FSVMTSTELLQSSAVAVTFGDRVLYPLSWIVPVFVVCSTFGAANGSCFTAGRLTYVAGREGHMVKIMSYI

SVKRYTPSPALMFNGIVSIIYIMPTDINTLINYFSFATWLFYGLTCLALIVMRFTRKDLKRPVKVPIVIP

ALVVVVSCYLVLAPIIDKPEWEYLYCTMFIVGGLLLYVPFIHYKFNWTRRLMRPFTMHLQLLLQVVPPEK

IE

>medaka_Slc7a9|ENSORLP00000014785|H2M8G9 (H2M8G9_ORYLA)

MGEGSIKKRQPHNGTTGPTEKEKTEKATALQKDVGLLSGICLIVGTMIGSGIFISPKSVLLYSGSVGPCL

LIWTACGVLSTLGALCYAELGTMITKSGGEYPYLMEAFGSIVAYLYSWTTVIVLKPSSFAIIALSFAEYT

ATPFYPGCTPPTVIIKCLAIASLLIIVAINCISVKLASYVQNFFTAAKLVIILVIVVAGIVLLAQGKTQN

LSNAFDGSSMSFGAIGLAFYNGLWAYDGWNQLNFITEELRNPYRNLPLAIIIGIPLVTVCYVLVNIAYFT

VLTPSELLVSPAVAVTFGDRVLYPLSWIVPLFVAFSTFGAANGSCFTAGRLAFVSGREGHMVKILSYVSL

RRYTPSPALIFNGILSLFYIIPADINSLINYFSFAQWAFYGLTALSLIVMRFTRKELHRPVRVPIVIAAL

IVVVSCYLVLAPIIDQPELEYLYCTIFIFSGLILYYPFVYLKPRWARRIMRPITMHLQLFMEVVPPEDYE

PEDPMKSVKF

>fugu_rubripes_Slc7a9|ENSTRUP00000000655|H2RKJ2 (H2RKJ2_TAKRU)|see_also_XP_011616887.1

MDKESIKERKESQNGSTGQTTDSQTKDGLAEKATVLQKNVGLLSGICLIVGTIIGSGIFISPKAVLLYSG

AVGPCLLIWASCGVLSILGALCYAELGTTIVKSGGDYSYYLEAFHPIVAFLFSWTTVIALKPSSLAIITL

SFAEYASSPFFPGCSPPTIVIKFLAATAILLIVTVNSLSVTLANYVQNFFTAAKLFIILVIAIAGIVLLA

QGKTENLSNAFDGASTSFGAIGLAFYNGFWAYDGWNQLNFITEELKNPHRNLPLAILIGISLVTVCYVLV

NVAYFTVMTPSELLLSPAVAITFGDRVFYPLSWVVPLFVAFSTFGAANGSCFTAGRISYVSSREGHMVQI

LSFISLKHCTPSPAIIFNGLLAICYIIPADIGILINYFSFAQWGFYGMSALALIVMRFTRKDLHRPVKVP

IVLAFLLGLLSCYLVLAPIIDKPTIEYLYCSIFIFSGVILYYFFIHRKVKWAQRVSRPITTYLQLLMEVV

PPEKTETD

>spotted_green_pufferfish_Slc7a9|ENSTNIP00000007317|H3CGD9 (H3CGD9_TETNG)|see_also_ENSTNIP00000000982|H3BYB6 (H3BYB6_TETNG)

QAQKEPQNDSTDQTTGSQTKNKAPERAAALQKNVGLFSGTCLIVGTIIGSGIFISPKAVLLYSGAVGPCL

LIWAACGVLSILGALCYAELGTTITKSGGDYSYYLEAFHPIVAFLFSWTMVIVLKPSSLAIITLSFAEYV

SSPFYPGCSPPIIITKFLAATAILLIVTVNSLSVRLASYVQNFFTTAKLLIIFVIVIAGVVMLAQGKTQN

LSNAFEGASTSFGAIGLAFYNGFWAYDGWNQLNFITEELKNPHRNLPLAILIGISLVSVCYVLVNVAYFT

VMTPSELLLSPAVAVTFGDRVFYPLSWIVPLFVAFSTFGAANGSCFTAGRLSYVSSREGHMVQILSFISL

KHCTPSPAIIFNGLLAICYIIPADIGTLINYFSFAQWGFYGMSALALIVLRFTRKDLHRPVKVPIVLAFL

LALVSCYLVLAPIIDKPTIEYLYCSIFIFSGVILYYFFIYRKVKWAQRISRVITTYLQLLMEVVPPEETK

T
