# Supplementary material for: The zebrafish cationic amino acid transporter/glycoprotein-associated family: sequence and spatiotemporal distribution during development of the transport system b0,+ (slc3a1/slc7a9)
Source: Fish Physiol Biochem. 2021 Aug 2;47(5):1507–25. doi: 10.1007/s10695-021-00984-z (PMC8478756; doi:10.1007/s10695-021-00984-z)
Supplement: Supplementary file 2 — Supplementary file2 (DOCX 615 KB) [file 10695_2021_984_MOESM2_ESM.docx]

**The zebrafish cationic amino acid transporter/glycoprotein-associated family: sequence and spatiotemporal distribution during development of the transport system b^0,+^ (*slc3a1*/*slc7a9*)**

Ståle Ellingsen, Shailesh Narawane, Anders Fjose, Tiziano Verri, Ivar Rønnestad

**Supplementary Materials II**

**Materials and Methods**

**Synopsys of the preliminary (function) results**

**Fig. R1**

**Fig. R2**

**Materials and Methods**

*Morpholino injections*

Morpholino antisense oligonucleotide *slc7a9_E2I2*: 5’-GATCTAGACTTCTACTTACAGATGC-3’ and a control mismatch oligonucleotide: 5’-GATTTAAGGCATAGCTTGCAATCCT-3’ were purchased from Gene Tools, LLC (<http://www.gene-tools.com/>). The morpholino *slc7a9 E2I2* was designed to hybridize to the exon/intron boundary at exon 2 and intron 2 of the *slc7a9* gene. Morpholino was resuspended in water at 2 mM and 1 mM concentration. Zebrafish embryos (wild type and NG43 enhancer trap line expressing GPF in the entire nephron and floor plate) were injected at one-cell stage with an approximately 2 nl (20 ng/10 ng) volume of 2 mM/1 mM morpholino (heated for 10 min at 65 °C, briefly vortexed) by using a microinjector (Picospitzer III, General Valve Corp., Fairfield, NJ). Embryos were observed using Zeiss SteREO Lumar.V12^TM^ microscope (Carl Zeiss, Germany) at 24 hpf, 48 hpf, 3 dpf, 4 dpf (shown in **Fig. R1**) and 5 dpf. GFP-fluorescence images were captured using Zeiss Axio Observer A1^TM^ inverted fluorescent microscope (Carl Zeiss, Germany) and bright field images were captured using Leica M420^TM^ stereo-microscope (Leica Microsystems, Heerbrugg, Switzerland).

*Verification of slc7a9 gene knockdown by morpholino slc7a9_E2I2*

Total RNA was isolated from 2 dpf control and phenotypic morphant embryos using TRIZOL (Boehringer, Mannheim, Germany) according to the manufacturer’s instruction. The total RNA was treated with Dnase I prior to final purification step. The first-strand cDNA was synthesized from 1 µg total RNA using M-MLV Reverse Transcriptase (Promega, Madison, USA) according to the protocol supplied. The second-strand synthesis was performed using standard PCR protocols with *slc7a9*-specific primers and Platinum Taq polymerase (Invitrogen, Carlsbad, USA). The verification of the knockdown effect of morpholino slc7a9_E2I2 was done by PCR (shown in **Fig. R2**) using the *slc7a9*-specific primers. The following (Morphocheck) primers *slc7a9 MorCHK_F*: 5’-GTGGGTCCATGTCTTTGTGTGT-3’ and *slc7a9 MorCHK_R*:5’-ATAAGCCCAAAGACCGTTGTAAA-3’ located in the exons flanking the targeted exon/intron boundary were used to amplify fragments. These fragments were then gel extracted, cloned into pCRII-TOPO (Invitrogen, Carlsbad, USA) and sequenced. The sequences obtained were compared using ENSEMBL blast search to verify altered splicing.

**Synopsys of the preliminary (function) results**

*Heteromeric amino acid transporters appear to be needed for proper formation of the proximal convoluted tubule segment of the zebrafish nephron*

We studied the developmental role of system b^0,+^ in zebrafish by morpholino-mediated knockdown experiments. Since it was previously reported that: i) *slc3a1* is rapidly degraded when *slc7a9* is not present (Bartoccioni et al. 2008); ii) human embryonic kidney 293 cells show that ER-Golgi trafficking of *slc3a1/slc7a9* heterodimer is inhibited if the light chain partner (i.e. *slc7a9)* is absent or is truncated (Sakamoto et al. 2009), we designed a morpholino targeting the *slc7a9* gene to obtain a collective inhibition of the heterodimer subunits (Slc3a1-Slc7a9) in the cells where they are co-expressed.

To analyze the effects on nephron development, we injected the *slc7a9* morpholino into fertilized embryos from the enhancer trap line NG43, which expresses EGPF in the entire nephron (unpublished data). In this line, proper (convolution of the) proximal convoluted tubule of the nephron (PCT) could be seen up to 5 dpf, posterior to the neck (N) segment of the nephron (e.g. indicated by white arrowhead in **Fig. R1** is the neck of a nephron at 4 dpf, which is followed by the rest of the tubule). Another EGFP expressing tubule like layer of cells that emerged from the hindbrain and extended to the tail was the floor plate (FP) of the neural tube (the FP is seen between the two nephrons in the dorsal view and above the nephrons in the tilted/lateral view while the left nephron PCT is indicated by white arrowhead). In particular, in this preliminary analysis: i) we counted ~50% *slc7a9* morphant embryos with pericardial edema and slightly reduced yolk absorption, while the mismatch morpholino-injected control embryos were normal (Table R1); ii) *slc7a9* morphants showed delayed overall growth and reduction in the proximal nephron size during 24 hpf to 72 hpf; iii) all the *slc7a9* morphant embryos lacking the proximal tubule structure of the nephron also showed pericardial edema at 4 dpf (Table R1). Notably, this phenotype appeared similar to that of cardiac *tnnt2* and *ift88* morpholino-injected embryos lacking proximal tubule structures (Vasilyev et al. 2009).

**Fig. R1** is meant to summarize the observed results. In control embryos (see **Fig. R1A** and **Fig. R1C**), the nephron was properly developed at 4 dpf as compared to the *slc7a9* morphants (see **Fig. R1B** and **Fig. R1D**). At this developmental stage, *slc7a9* morphants showed a reduction of the proximal nephron EGFP that was also accompanied by pericardial edema (see **Fig. R1B** and **Fig. R1D**). Major phenotypic features of *slc7a9* morphants were either PCT inhibition where morphants lacked the convolution of the proximal segment of the nephron indicated by white arrowhead (**Fig. R1B**) or the PCT deformation where the convolutions were not proper as compared to that of the wild type (data not shown). Lack of proper proximal tubule segmentation could be explained by an impaired glomerular filtration and impaired osmotic regulation. Such kidney malfunction in a freshwater living teleost might have caused water retention that generated the observed pericardial edema (PE) (dark arrowhead, **Fig. R1D**).

To help understand and compare the structural and segmental organization of the zebrafish nephron, a schematics of normal nephron segmentation is depicted at the bottom of the figure (pictures and terminology modified from Wingert et al. 2007). The schematic of the *slc7a9* morphant that lacked the PCT and the proximal structures of the nephron is depicted aside.

Verification of morpholino mediated alteration in exon/intron splicing was revealed by band shift in RT-PCR (**Fig. R2**) and was further confirmed by sequencing and blast alignment.


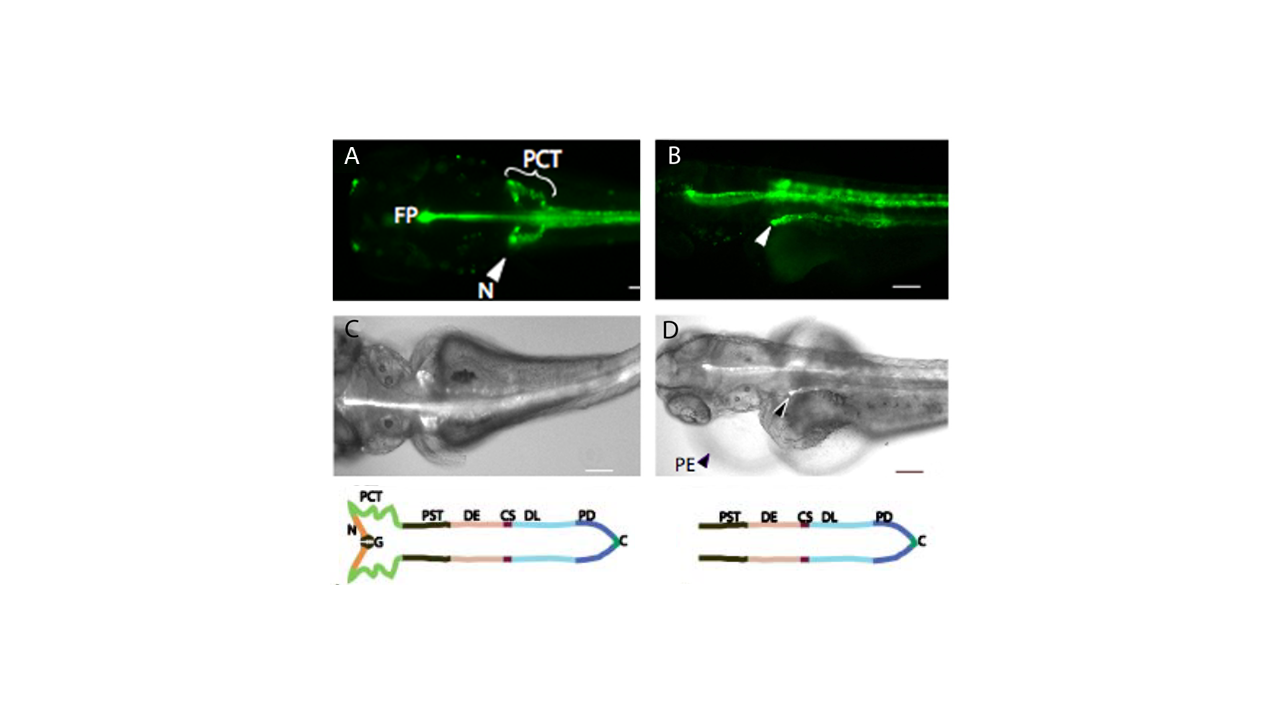


**Fig. R1. Knockdown analysis in the wild type and NG43 enhancer trap line using *slc7a9*-based morpholino approach.** GFP expression is seen in the nephron (N) and a floor plate (FP) in the control injected NG43 4 dpf embryos (**A**, white arrowhead indicated the position of PCT). *slc7a9* morphants show (**B**) PCT inhibition (65%, n=19) (white arrowhead). Fusion of the GFP (white channel) and bright field images (**C**, normal and (**D**, *slc7a9* morphants) show the pericardial edema (PE, dark arrowhead) and PCT inhibition (dark arrowhead) in the morphant embryos (**D**). Schematics of the zebrafish nephron (modified from Wingert et al 2007). Abbreviations: glomerulus (G), neck (N), proximal convoluted tubule (PCT), proximal straight tubule (PST), distal early (DE), corpuscle of Stannius (CS), distal late (DL), pronephric duct (PD) and the cloaca (C) (Scale bar 200 μm).

**A
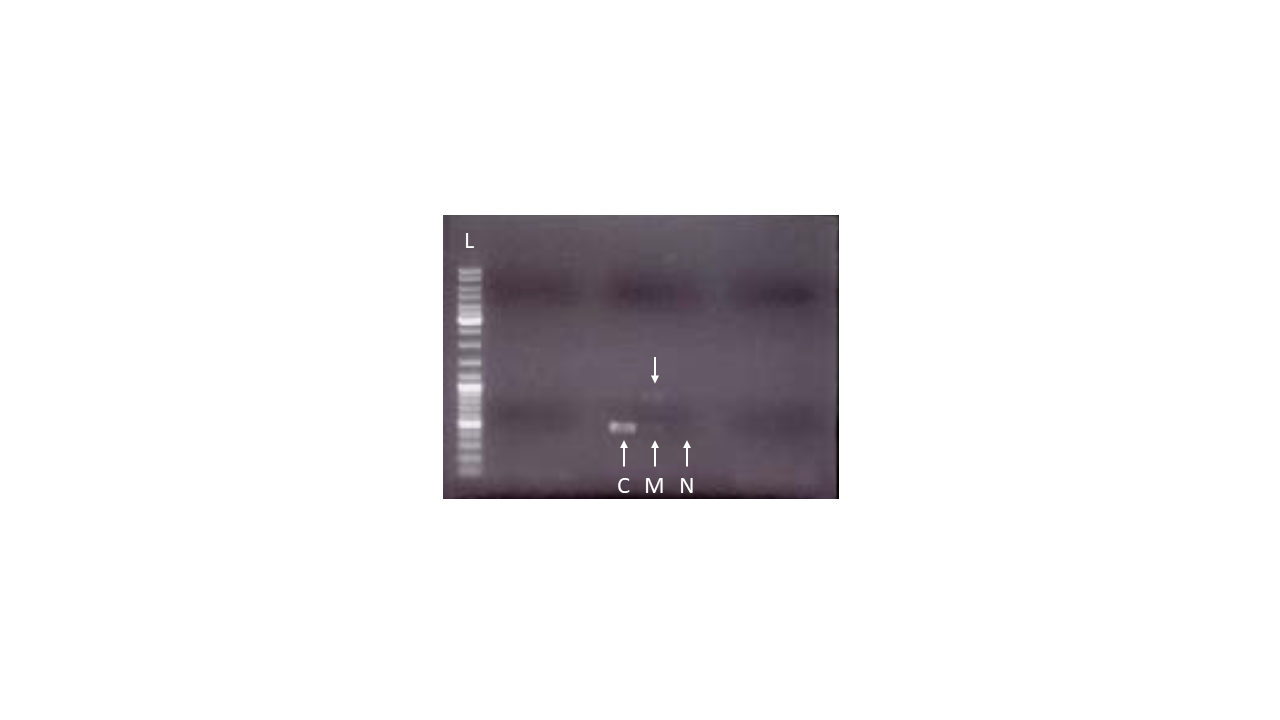
 B
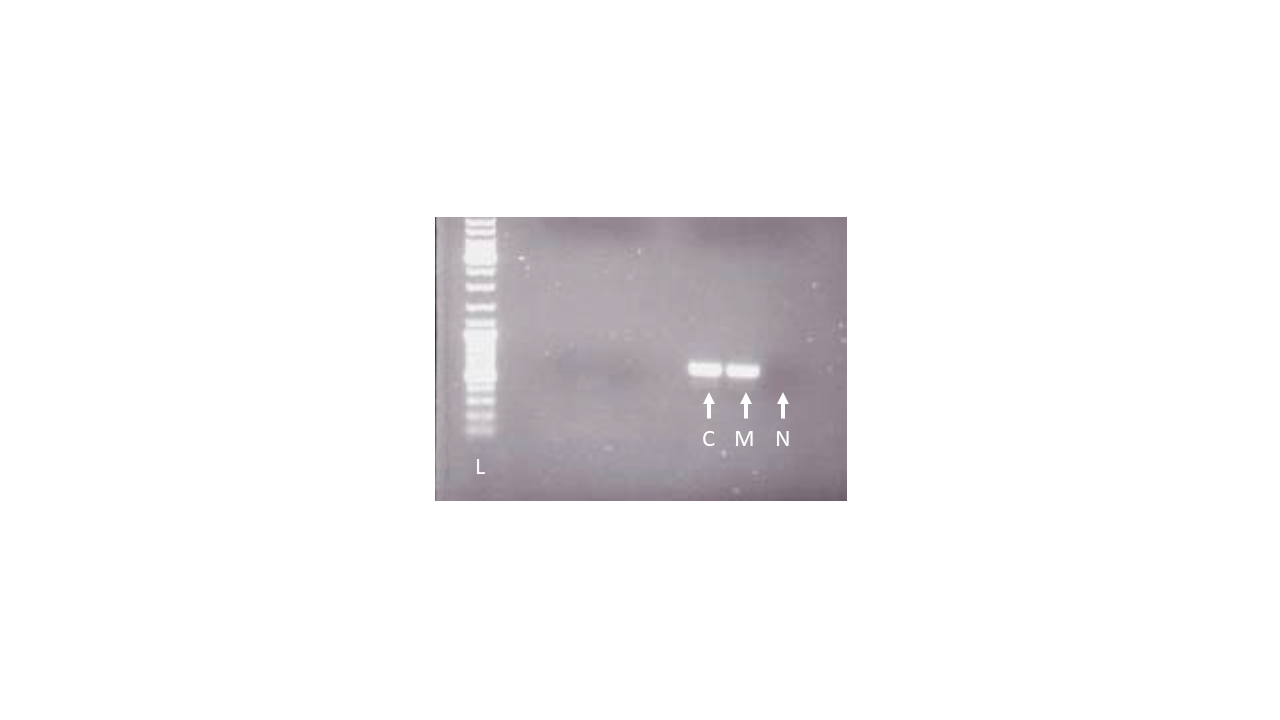
**

**Fig. R2. RT-PCR to check splice blocking by morpholino *slc7a9_E2I2*.** (**A**) RT-PCR with primers *slc7a9 MorCHK_F*: and *slc7a9 MorCHK_R* (see ‘*Morpholino injections*’ above). (**B**) RT-PCR with β-actin primers to control for RT-PCR integrity and template concentrations.

C, positive control, normal embryo; M, positive control, *slc7a9* morphants with phenotype; N, negative control (no template).

Table R1. slc7a9 morpholino injection statistics

| Condition | ng/embryo | % Pericardial edema | % PCT inhibition | % PCT  Deformation | % Normal | Total#* |
| --- | --- | --- | --- | --- | --- | --- |
| Uninjected | 0 | 0 | 0 | 0 | 100 | 120 |
| Mismatch control | 20 | 0 | 0 | 0 | 98 | 86 |
| Slc7a9MO | 20 | 50 | 65 | 35 | 50 | 60 |

*Combined total from 3 independent experiments

Legend

ng/embryo = nanograms of reagent injected per embryo

% PCT inhibition = percent of NG43 embryos showing PCT inhibition along with pericardial edema

% PCT deformation= percent of NG43 embryos showing PCT deformation along with pericardial edema

% Normal = percent of embryos with no pericardial edema or defect in PCT

Total # = number of embryos surviving past gastrulation

**References**

Bartoccioni P, Rius M, Zorzano A, Palacín M, Chillarón J (2008) Distinct classes of trafficking rBAT mutants cause the type I cystinuria phenotype. Hum Mol Genet 17:1845-1854.

Sakamoto S, Chairoungdua A, Nagamori S, Wiriyasermkul P, Promchan K, Tanaka H, Kimura T, Ueda T, Fujimura M, Shigeta Y, Naya Y, Akakura K, Ito H, Endou H, Ichikawa T, Kanai Y (2009) A novel role of the C-terminus of b0,+ AT in the ER-Golgi trafficking of the rBAT-b 0,+ AT heterodimeric amino acid transporter. Biochem J 417:441-448.

Vasilyev A, Liu Y, Mudumana S, Mangos S, Lam PY, Majumdar A, Zhao J, Poon KL, Kondrychyn I, Korzh V, Drummond IA (2009) Collective cell migration drives morphogenesis of the kidney nephron. PLoS Biol 7:e9.

Wingert RA, Selleck R, Yu J, Song HD, Chen Z, Song A, Zhou Y, Thisse B, Thisse C, McMahon AP, Davidson AJ (2007) The cdx genes and retinoic acid control the positioning and segmentation of the zebrafish pronephros. PLoS Genet 3:1922-1938
